# *ORMDL3* expression in ASM regulates hypertrophy, hyperplasia via TPM1 and TPM4, and contractility

**DOI:** 10.1172/jci.insight.136911

**Published:** 2021-04-08

**Authors:** Alexa K. Pham, Marina Miller, Peter Rosenthal, Sudipta Das, Ning Weng, Sunghoon Jang, Richard C. Kurten, Jana Badrani, Taylor A. Doherty, Brian Oliver, David H. Broide

**Affiliations:** 1 Department of Medicine, University of California San Diego, La Jolla, California, USA.; 2 Department of Pediatrics, Arkansas Children’s Research Institute, College of Medicine, University of Arkansas for Medical Sciences, Little Rock, Arkansas, USA.; 3 Veterans Affairs San Diego Health Care System, La Jolla, California, USA.; 4 School of Life Sciences, University of Technology Sydney, Sydney, Australia.

**Keywords:** Muscle Biology, Pulmonology, Asthma

## Abstract

ORM1-like 3 (*ORMDL3*) has strong genetic linkage to childhood onset asthma. To determine whether *ORMDL3* selective expression in airway smooth muscle (ASM) influences ASM function, we used Cre-*loxP* techniques to generate transgenic mice (*hORMDL3*^Myh11eGFP-cre^), which express human ORMDL3 selectively in smooth muscle cells. In vitro studies of ASM cells isolated from the bronchi of *hORMDL3*^Myh11eGFP-cre^ mice demonstrated that they developed hypertrophy (quantitated by FACS and image analysis), developed hyperplasia (assessed by BrdU incorporation), and expressed increased levels of tropomysin proteins TPM1 and TPM4. siRNA knockdown of TPM1 or TPM4 demonstrated their importance to ORMDL3-mediated ASM proliferation but not hypertrophy. In addition, ASM derived from *hORMDL3*^Myh11eGFP-cre^ mice had increased contractility to histamine in vitro, which was associated with increased levels of intracellular Ca^2+^; increased cell surface membrane Orai1 Ca^2+^ channels, which mediate influx of Ca^2+^ into the cytoplasm; and increased expression of ASM contractile genes sarco/endoplasmic reticulum Ca^2+^ ATPase 2b and smooth muscle 22. In vivo studies of *hORMDL3*^Myh11eGFP-cre^ mice demonstrated that they had a spontaneous increase in ASM and airway hyperreactivity (AHR). *ORMDL3* expression in ASM thus induces changes in ASM (hypertrophy, hyperplasia, increased contractility), which may explain the contribution of *ORMDL3* to the development of AHR in childhood onset asthma, which is highly linked to *ORMDL3* on chromosome 17q12-21.

## Graphical abstract

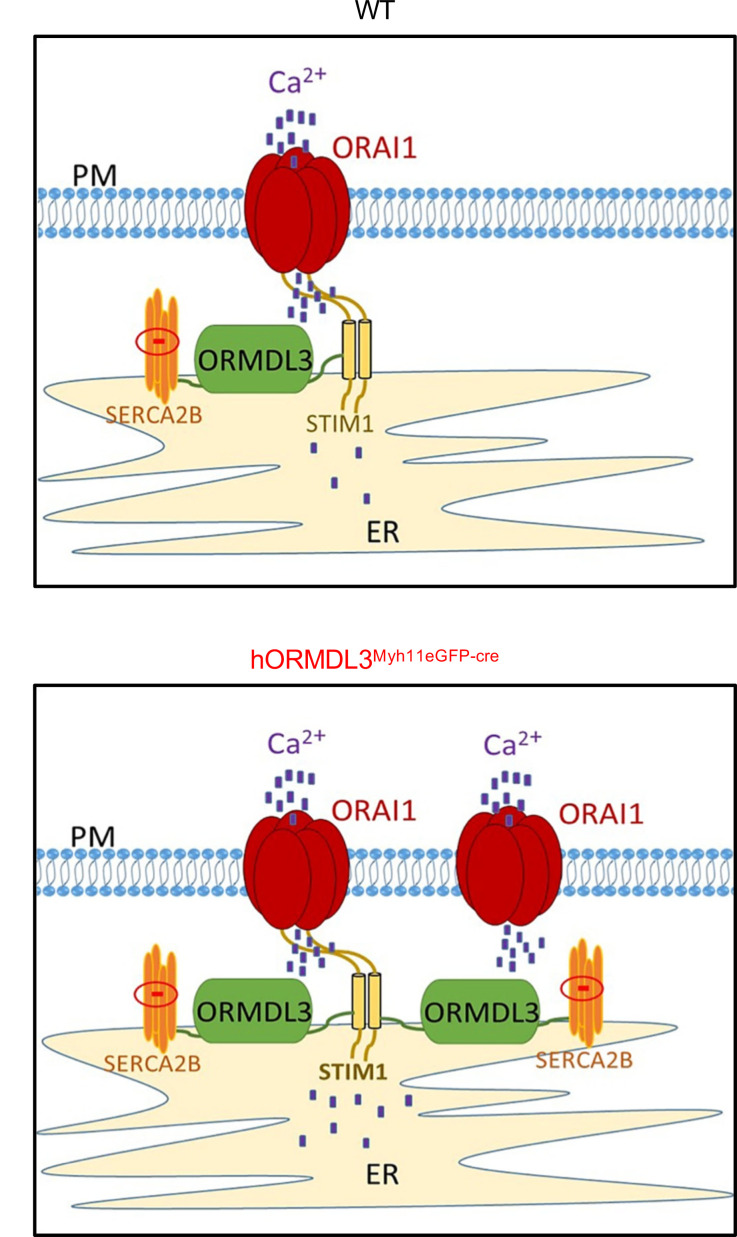

## Introduction

ORM1-like 3 (*ORMDL3*), a gene located on chromosome 17q12-21, has been highly linked to childhood onset asthma in genetic association studies (1). *ORMDL3* is an ER-localized protein (2) expressed in multiple cell types important to the pathogenesis of asthma, including immune and inflammatory cells [i.e., CD4^+^ cells (3, 4), eosinophils (5), and macrophages (2)] and lung structural cells [i.e., epithelial cells (2) and airway smooth muscle (ASM) (6)]. At present it is not known which of these or other cells alone or in combination express the key *ORMDL3*-regulated pathways important to the development of asthma. In terms of immune and inflammatory cells expressing *ORMDL3*, CD4^+^ cells expressing SNPs linked to *ORMDL3* have enhanced Th2 responses (4), suggesting that *ORMDL3*-regulated pathways in CD4^+^ cells may be the key cell implicating *ORMDL3* in asthma. However, epidemiologic studies demonstrate that chromosome 17q12-21 is linked to childhood asthma (1) but not to allergic rhinitis (7). As both childhood asthma and allergic rhinitis are associated with increased Th2 responses, one would have expected that *ORMDL3* enhancing Th2 responses would result in association of *ORMDL3* with both childhood asthma and allergic rhinitis, which is not the case. Thus, *ORMDL3* expression in CD4^+^ cells may be very important for enhancing Th2 responses in childhood asthma but may not be the key cell expressing ORMDL3, implicating it in the onset of asthma in childhood.

One of the other key cells that is present in the lung in asthma, but not present in the upper airway in allergic rhinitis, is ASM, whose expression of *ORMDL3* may help explain the epidemiologic association of chromosome 17q21-21 with childhood onset asthma but not allergic rhinitis. To determine the role of selective *ORMDL3* expression in ASM, in this study we have used Cre-*loxP* techniques to generate mice expressing human *ORMDL3* (*hORMDL3*) selectively in smooth muscle cells. These studies demonstrated that ASM cells isolated from the bronchi of *hORMDL3*^Myh11eGFP-cre^ (myosin heavy chain 11, Myh11) mice spontaneously developed hypertrophy, hyperplasia (mediated by tropomysins TPM1 and TPM4), and increased contractility to histamine in vitro, which was associated with increased levels of intracellular Ca^2+^, increased cell surface membrane Orai1 Ca^2+^ channels — which mediate influx of Ca^2+^ into the cytoplasm — and increased expression of ASM contractile genes, including sarco/endoplasmic reticulum Ca^2+^ ATPase 2b (Serca2b) and smooth muscle 22 (Sm22). In vivo studies of *hORMDL3*^Myh11eGFP-cre^ mice demonstrated that they had a spontaneous (in the absence of experimental exposure to allergen or other environmental trigger) increase in ASM, which was associated with a spontaneous increase in airway hyperreactivity (AHR). *ORMDL3* expression in ASM thus induces changes in ASM (hypertrophy, hyperplasia, and increased contractility), which may explain the contribution of *ORMDL3* to the development of AHR in childhood onset asthma but not to the development of allergic rhinitis.

## Results

### hORMDL3^Myh11eGFP-cre^ mouse ASM cells for in vitro study.

**Figure 1:**
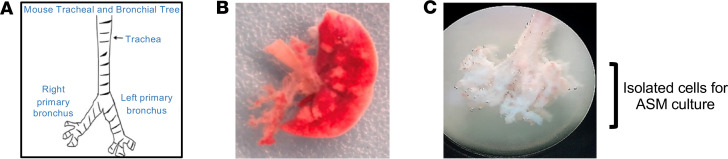
Mouse ASM cells. (**A**) The mouse tracheobronchial tree was dissected free from (**B**) whole lung tissue using (**C**) a dissecting microscope under 10× objective, and a single-cell suspension of bronchial cells was prepared for culture in mouse smooth muscle cell media.

**Figure 2:**
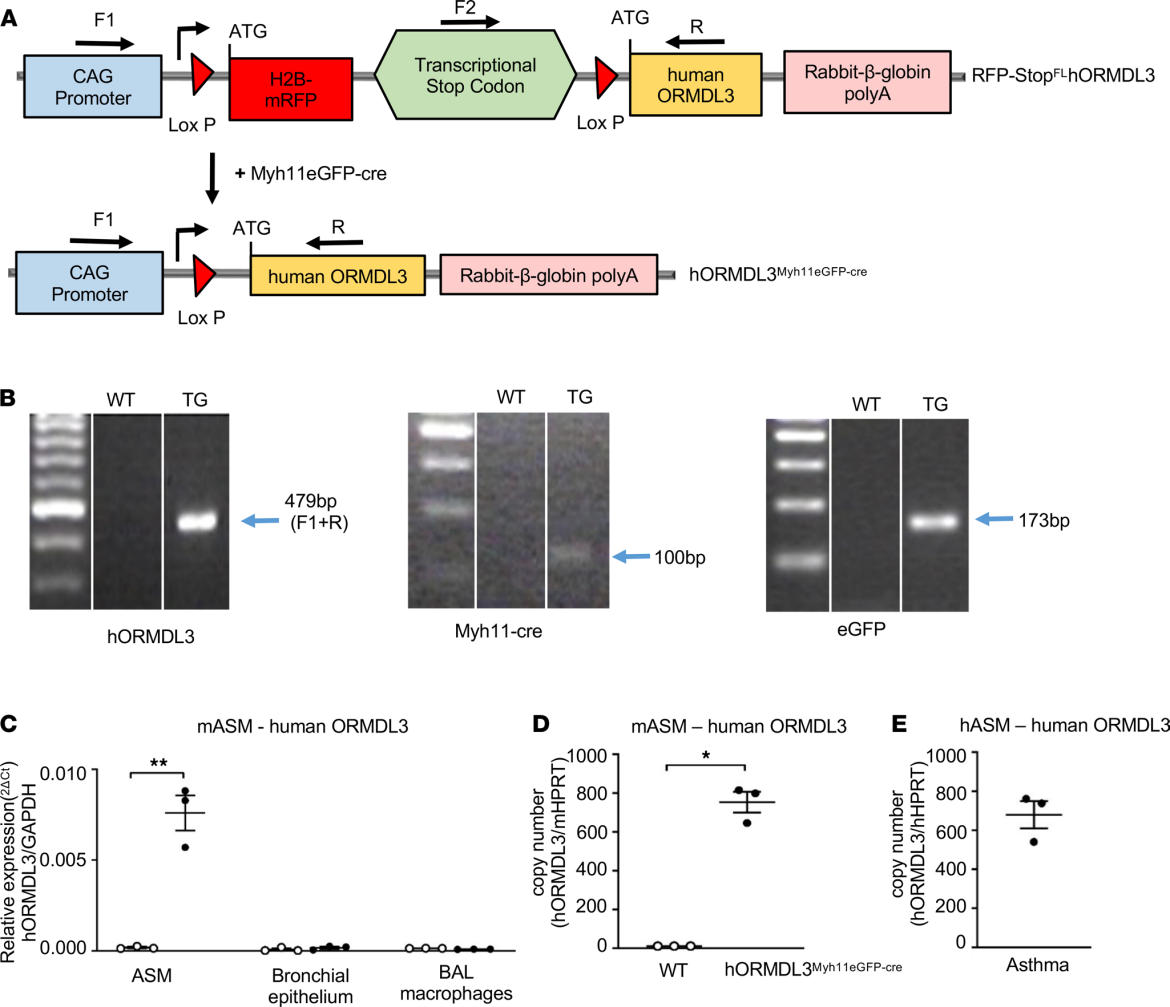
Genotyping and *hORMDL3* expression in ASM from mice and asthmatics. (**A**) RFP-Stop^fl^*hORMDL3*-Tg mice were crossed with smooth muscle–specific Myh11-creEGFP mice to generate *hORMDL3*^Myh11eGFP-cre^ (both on C57BL/6 background), which expresses *hORMDL3* specifically in smooth muscle cells. (**B**) WT and *hORMDL3*^Myh11eGFP-cre^ mice were genotyped to determine expression of human *ORMDL3*, Myh11-Cre, and EGFP. (**C**) Human *ORMDL3* mRNA expression was quantitated by RT-qPCR in mouse ASM, mouse bronchial epithelium, and mouse bronchoalveolar lavage (BAL) macrophages derived from WT and *hORMDL3*^Myh11eGFP-cre^ mice (*n* = 3/group). (**D**) Human *ORMDL3* mRNA copy number was quantitated by dPCR in mouse ASM derived from WT and *hORMDL3*^Myh11eGFP-cre^ mice (*n* = 3/group). (**E**) Human *ORMDL3* mRNA copy number was quantitated by digital PCR (dPCR) in human asthma postmortem lung ASM (*n* = 3) *P < 0.05 compared with WT. **P < 0.01 compared with WT. *P* values are results of unpaired 2-tailed *t* tests.

Mouse ASM cells were obtained from the bronchial airways dissected free of lung tissue of *hORMDL3*^Myh11eGFP-cre^ and WT mice (Figure 1, A–C). Bronchial airway cells were cultured in mouse smooth muscle cell media (Cell Biologics) for 3 to 4 weeks at which time 90% of the cells were ASM cells as defined by expression of ASM genes (alpha–smooth muscle actin, myh11) (Figure 2, A and B), expression of EGFP (Figure 2, A and B), and morphology.

### Generation of hORMDL3^Myh11eGFP-cre^ Tg mice.

To generate *hORMDL3*^Myh11eGFP-cre^ mice, we crossed RFP-Stop^fl^*hORMDL3*-Tg mice (8) (which have a floxed transcriptional stop codon positioned in front of the *hORMDL3* transgene, preventing *hORMDL3* transcription) with a smooth muscle–specific cre mouse (Myh11-creEGFP) (cre expressed selectively in smooth muscle cells under the smooth muscle myosin heavy chain Myh11 promoter tagged with EGFP) (9) (Figure 2A). Crossing the RFP-Stop^FL^*hORMDL3*-Tg mice with Myh11-creEGFP mice results in smooth muscle–expressed cre excising the floxed transcriptional stop codon positioned in front of the *hORMDL3* transgene, which then allows *hORMDL3* expression in smooth muscle cells in *hORMDL3*^Myh11eGFP-cre^ but not littermates (referred to as WT) (Figure 2A).

### Selective expression of hORMDL3 in ASM derived from hORMDL3^Myh11eGFP-cre^ mice.

Primer sets detected predicted sizes of mRNA by reverse transcription quantitative PCR (RT-qPCR) for *hORMDL3*, Myh11-Cre, and *eGFP* in progeny of RFP-Stop^fl^*hORMDL3*-Tg mice crossed with Myh11-cre*eGFP* mice (i.e., *hORMDL3*^Myh11eGFP-cre^ or WT littermate mice) (Figure 2B). The *hORMDL3* transgene in *hORMDL3*^Myh11eGFP-cre^ mice was selectively expressed in smooth muscle cells and was not detected in airway epithelium (Figure 2C), or in BAL macrophages (Figure 2C), as assessed by RT-qPCR. Significant expression of the *ORMDL3* transgene was detected in ASM derived from *hORMDL3*^Myh11eGFP-cre^ mice but not in ASM derived from WT mice (Figure 2C). We also quantitated *hORMDL3* mRNA copy number in ASM derived from *hORMDL3*^Myh11eGFP-cre^ mice compared with WT mice. Copies of *hORMDL3* mRNA were readily detected in ASM derived from *hORMDL3*^Myh11eGFP-cre^ mice but not in ASM derived from WT mice (Figure 2D). Additionally, copies of *hORMDL3* mRNA in human ASM from asthmatic airways showed relatively similar *ORMDL3* copy number (Figure 2E) as compared with *ORMDL3* copy number in ASM derived from *hORMDL3*^Myh11eGFP-cre^ mice (Figure 2D). Expressing human *ORMDL3* in ASM in *hORMDL3*^Myh11eGFP-cre^ mice did not influence levels of endogenous murine Ormdl3 expression in lung ASM (Supplemental Figure 1; supplemental material available online with this article; https://doi.org/10.1172/jci.insight.136911DS1).

### Increased ASM hypertrophy in hORMDL3^Myh11eGFP-cre^ mice.

**Figure 3:**
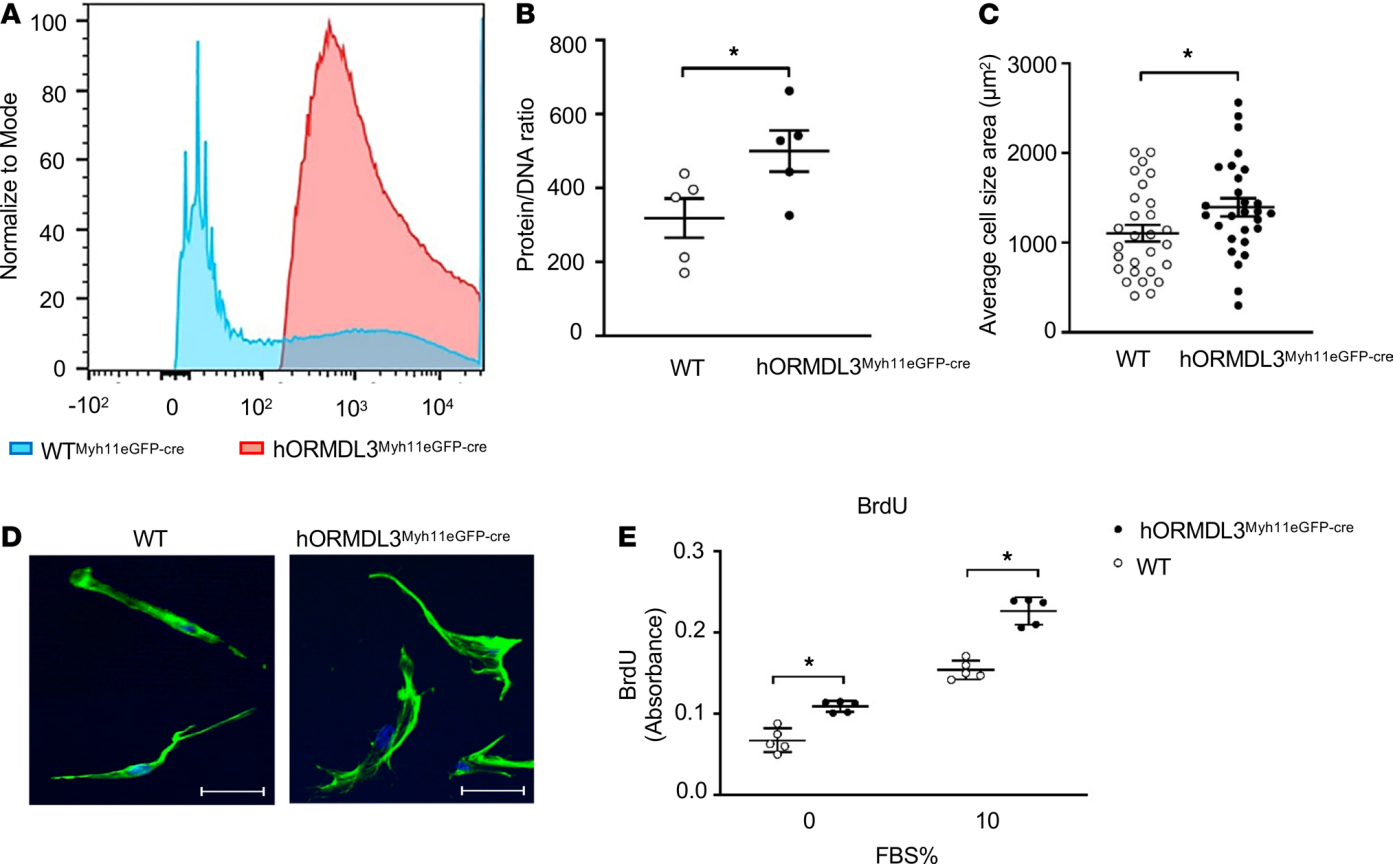
Effect of *ORMDL3* on ASM hypertrophy and hyperplasia. (**A**) FACS analysis using forward scatter plot of ASM from WT (blue peak) and *hORMDL3*^Myh11eGFP-cre^ (red peak) mice was used to determine ASM cell size after gating on live ASM cells. (**B**) Protein/DNA ratio was used as an index of ASM hypertrophy (*n* = 5/group). (**C** and **D**) Microscopic image analysis of ASM quantitated average cell size area following staining with phalloidin tagged with Alexa Fluor 488 (*n* = 28/group). Scale bar: 50 μm. (**E**) ASM proliferation was determined by BrdU incorporation at baseline and following 10% FBS stimulation (*n* = 5/group). *P < 0.05 compared with WT. *P* values are results of unpaired 2-tailed *t* tests.

As ASM hypertrophy is a feature of asthma (10–12), we investigated whether *ORMDL3* expression in ASM influenced hypertrophy by measuring both cell size by FACS and ASM protein to DNA ratio (an index of cell hypertrophy) and by image analysis in ASM derived from *hORMDL3*^Myh11eGFP-cre^ and WT mice. ASM cells from *hORMDL3*^Myh11eGFP-cre^ mice were significantly larger in cell size compared with ASM from WT mice (*P* < 0.05) as quantitated by FACS (Figure 3A). The ratio of protein to DNA content in *hORMDL3*^smgfp-cre^ (smooth muscle green fluorescent protein, smgfp) ASM cells were also significantly higher compared with WT ASM cells (*P* < 0.05) (Figure 3B). In addition, ASM cells from *hORMDL3*^Myh11eGFP-cre^ mice were significantly larger in cell size compared with ASM from WT mice (*P* < 0.05) as quantitated by image analysis (Figure 3, C and D).

### Increased ASM proliferation in hORMDL3^Myh11eGFP-cre^ mice.

As ASM hyperplasia is a feature of asthma (10, 13, 14), we investigated whether increased expression of *ORMDL3* in ASM influenced proliferation utilizing a BrdU assay. Unstimulated *hORMDL3*^Myh11eGFP-cre^ ASM cells were significantly more proliferative compared with unstimulated WT ASM cells (*P* < 0.05) (Figure 3E). In addition, when stimulated to proliferate with 10% FBS, *hORMDL3*^Myh11eGFP-cre^ ASM cells were significantly more proliferative compared with 10% FBS–stimulated WT ASM cells (*P* < 0.05) (Figure 3E).

### Increased expression of TPM1 and TPM4 in ASM of hORMDL3^Myh11eGFP-cre^ mice.

**Figure 4:**
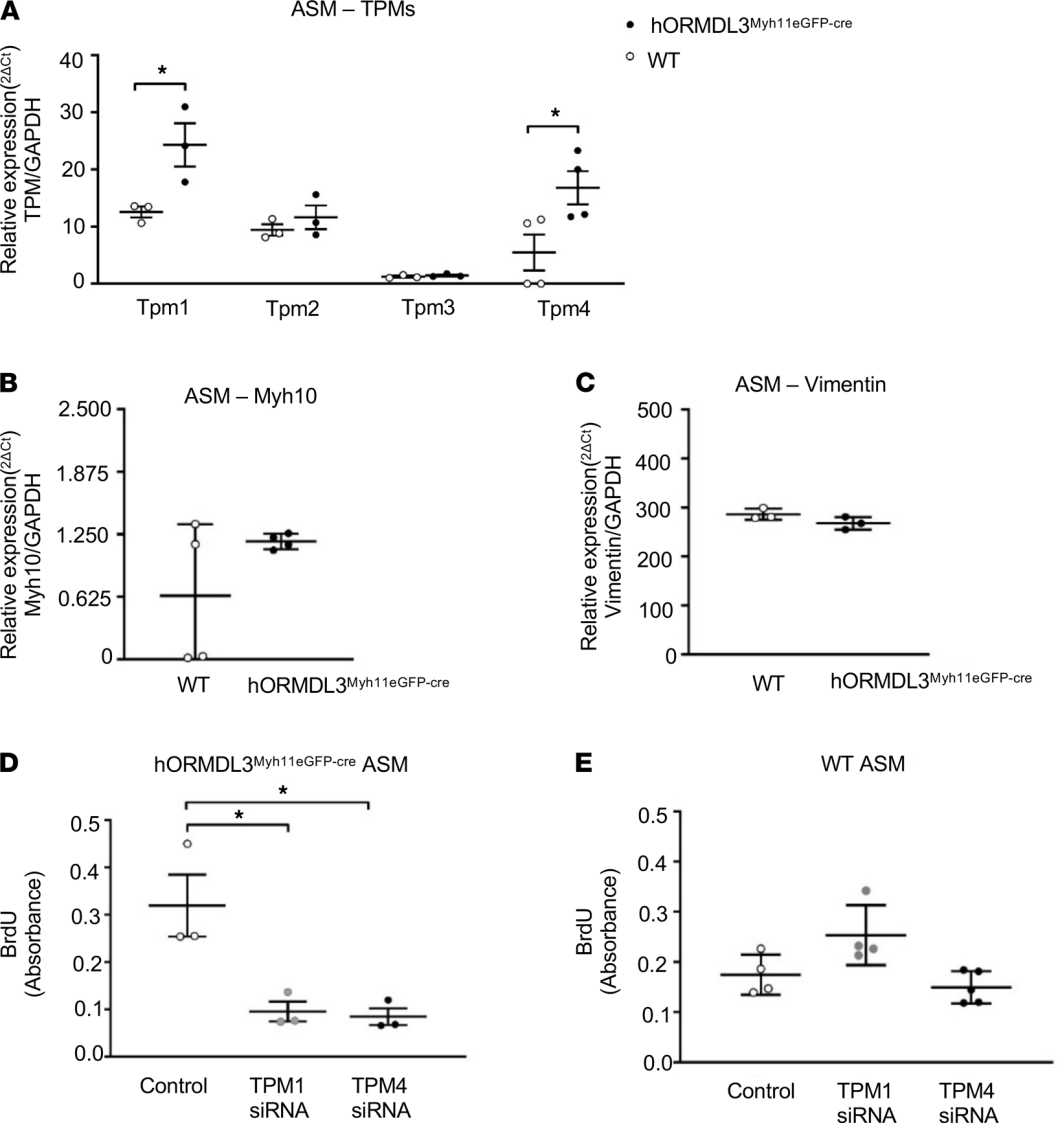
Effect of *ORMDL3* on ASM TPM expression and ASM proliferation. (**A**). Levels of TPM1, TPM2, TPM3, and TPM4 mRNA were quantitated by RT-qPCR in ASM derived from WT and *hORMDL3*^Myh11eGFP-cre^ mice (*n* = 3–4/group). (**B**) Levels of Myh10 and (**C**) vimentin mRNA were quantitated by RT-qPCR in ASM derived from WT and *hORMDL3*^Myh11eGFP-cre^ mice (*n* = 3–4/group). (**D**) BrdU proliferation assay of ASM from *hORMDL3*^Myh11eGFP-cre^ (*n* = 3/group) or (**E**) ASM from WT mice (*n* = 3/group) knocked down with TPM1 siRNA, TPM4 siRNA, or control scrambled siRNA. *P < 0.05 compared with control scrambled siRNA. *P* values are results of unpaired 2-tailed *t* tests (**A**–**C**) or 1-way ANOVA with Tukey’s post hoc test (**D** and **E**).

As ASM derived from *hORMDL3*^Myh11eGFP-cre^ mice had increased proliferation, we examined whether *ORMDL3* expressing ASM had markers associated with a proliferative synthetic ASM phenotype, including tropomysins 1–4, vimentin, and myosin heavy chain 10 (Myh10) (15). These studies demonstrated that ASM from *hORMDL3*^Myh11eGFP-cre^ mice had significantly higher levels of expression of TPM1 (*P* < 0.05) and TPM4 (*P* < 0.05) but not TPM2 or TPM3 mRNA by RT-qPCR compared with WT mice (Figure 4A). There was no increase in Myh10 (Figure 4B), vimentin (Figure 4C), or extracellular matrix proteins including laminin, perlecan, and fibronectin (Supplemental Figure 2A) in ASM from *hORMDL3*^smgfp-cre^ mice. Thus, *ORMDL3* upregulated a specific subset of ASM proliferative synthetic genes, in particular TPM1 and TPM4 (16). *ORMDL3* expression in ASM reduced expression of collagen type I and collagen type IV (Supplemental Figure 2A).

### TPM1 and TPM4 mediate ORMDL3-induced ASM proliferation.

As *ORMDL3* induced increased levels of TPM1 and TPM4 in ASM (Figure 4A), we investigated whether TPM1 and TPM4 regulate *ORMDL3*-induced ASM proliferation or hypertrophy by performing knockdown experiments using siRNAs for TPM1 or TPM4 in ASM. In ASM from *hORMDL3*^Myh11eGFP-cre^ mice siRNA knockdown of either TPM1 or TPM4 significantly reduced ASM proliferation compared with ASM transfected with scramble siRNA as assessed by BrdU incorporation (Figure 4D). In contrast, in WT ASM, which expressed significantly lower levels of TPM1 (*P* = 0.04) and TPM4 (*P* = 0.03) compared with ASM from *hORMDL3*^Myh11eGFP-cre^ mice, siRNA knockdown TPM1 or TPM4 in WT mice did not inhibit ASM proliferation (Figure 4E). Thus TPM1 and TPM4 do not contribute to baseline ASM proliferation in WT mice but do contribute to *ORMDL3*-induced ASM proliferation. The efficiency of TPM1 and TPM4 siRNA knockdown was approximately 50% and 80%, respectively.

Additionally, we investigated whether siRNA knockdown of TPM1 or TPM4 also regulated ASM hypertrophy by performing cell size measurement. In contrast to TPM1 and TPM4 regulating *ORMDL3*-induced ASM proliferation, siRNA inhibition of either TPM1 or TMP4 in ASM from *hORMDL3*^Myh11eGFP-cre^ or WT mice had no effect on ASM hypertrophy as quantitated by cell size using FACS (data not shown). This suggests that TPM1 and TPM4 play a significant role in *ORMDL3*-induced ASM hyperplasia but do not play a significant role in *ORMDL3*-induced ASM hypertrophy.

### ASM from hORMDL3^Myh11eGFP-cre^ mice have increased contractility to histamine in vitro.

**Figure 5:**
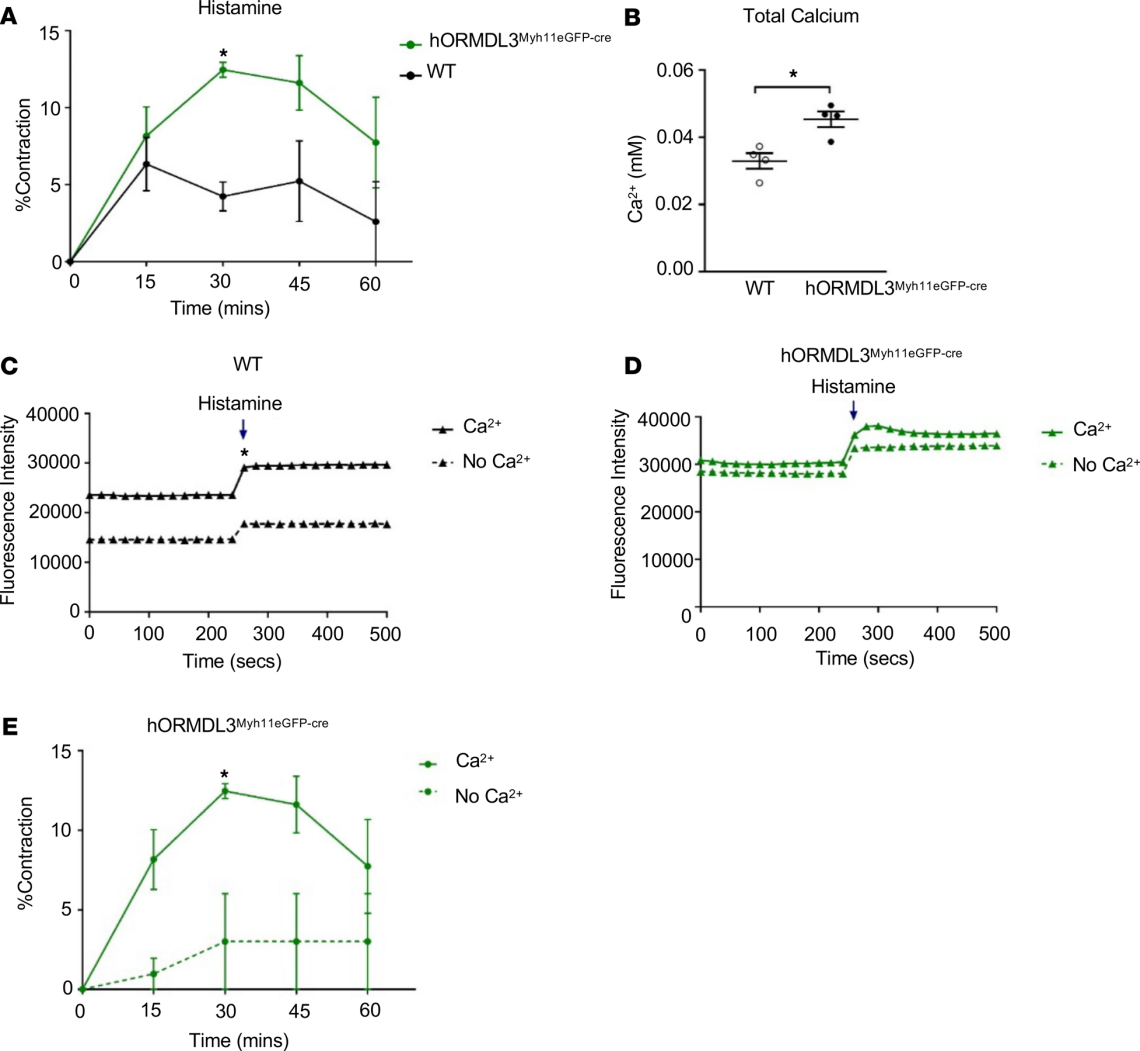
Effect of *ORMDL3* on ASM contractility and Ca^2+^ levels. (**A**) ASM contractility to histamine was quantitated in ASM from WT and *hORMDL3*^Myh11eGFP-cre^ mice (*n* = 3/group) by measuring the area of ASM-containing collagen gels. (**B**) Total intracellular Ca^2+^ levels (*n* = 4/group) and (**C** and **D**) calcium flux following 100 μm histamine stimulation were assessed in ASM from WT and *hORMDL3*^Myh11eGFP-cre^ mice in the presence or absence of extracellular Ca^2+^ (*n* = 3/group). (**E**) Contractility to histamine was quantitated in ASM-containing collagen gels from *hORMDL3*^Myh11eGFP-cre^ mice in the presence or absence of extracellular Ca^2+^ (*n* = 3/group). *P < 0.05 compared with WT or with no Ca^2+^. *P* values are results of unpaired 2-tailed *t* tests.

In these in vitro studies using an ASM gel contraction assay, ASM derived from the airways of *hORMDL3*^Myh11eGFP-cre^ mice had significantly increased contractility to histamine compared with WT ASM cells (*P* < 0.05) (Figure 5A).

### ASM from hORMDL3^Myh11eGFP-cre^ mice have increased intracellular Ca^2+^ levels and increased Ca^2+^ flux response to histamine.

As Ca^2+^ regulates ASM contraction by regulating the interaction of contractile proteins in ASM (17, 18), we investigated whether selective increased expression of *ORMDL3* only in ASM cells would influence not only ASM contractility (Figure 5A) but also ASM Ca^2+^ levels and flux. Interestingly, these studies demonstrated significantly higher baseline total free intracellular Ca^2+^ levels in *hORMDL3*^Myh11eGFP-cre^ ASM cells compared with WT ASM cells (*P* < 0.01) as assessed with a chromogenic calcium assay (Figure 5B).

To determine whether Ca^2+^ flux across the ASM cell surface plasma membrane was altered in ASM from *hORMDL3*^Myh11eGFP-cre^ mice, we measured Ca^2+^ flux in ASM cells loaded with the Ca^2+^-sensitive Fluo-4 Direct dye. Baseline levels of intracellular Ca^2+^ based on this dye indicator method were much higher in ASM derived from *hORMDL3*^Myh11eGFP-cre^ mice compared with WT ASM cells (*P* < 0.05) (Figure 5, C and D). However, ASM cells from *hORMDL3*^Myh11eGFP-cre^ and WT mice had a similar increase in Ca^2+^ influx following 100 μM histamine stimulation (Figure 5, C and D). Thus, 2 methods of measuring intracellular Ca^2+^, in which one utilizes a chromogenic assay (Figure 5B) and the second a dye indicator (Figure 5, C and D), both demonstrated that ASM derived from *hORMDL3*^Myh11eGFP-cre^ mice had increased intracellular Ca^2+^ levels. Overall, these results demonstrated that increased expression of *ORMDL3* in ASM leads to increased ASM contraction (Figure 5A), which is associated with increased baseline intracellular Ca^2+^ levels (Figure 5, C and D).

### ASM from hORMDL3^Myh11eGFP-cre^ mice has reduced requirement for extracellular Ca^2+^ for Ca^2+^ flux and contractile response to histamine.

We initially investigated whether removal of extracellular Ca^2+^ would affect the increased levels of intracellular Ca^2+^ in ASM from *hORMDL3*^Myh11eGFP-cre^ mice. Removal of extracellular Ca^2+^ did not significantly reduce the increased intracellular Ca^2+^ levels in ASM from *hORMDL3*^Myh11eGFP-cre^ mice (Figure 5D). In contrast, removal of extracellular Ca^2+^ significantly reduced the intracellular Ca^2+^ levels in ASM from WT mice (*P* < 0.05) (Figure 5C). As removal of extracellular Ca^2+^ did not significantly reduce the increased intracellular Ca^2+^ levels in ASM from *hORMDL3*^Myh11eGFP-cre^ mice (Figure 5D), we assessed whether removal of extracellular Ca^2+^ would affect the contractility of ASM from *hORMDL3*^Myh11eGFP-cre^ mice. Interestingly, ASM contraction to histamine in ASM derived from *hORMDL3*^Myh11eGFP-cre^ mice was significantly reduced in the absence of extracellular Ca^2+^ (Figure 5E). Taken together this suggests that although increased *ORMDL3* in ASM contributes to increased intracellular Ca^2+^ levels (even in the absence of extracellular Ca^2+^), extracellular Ca^2+^ levels play a crucial role in histamine-induced ASM contraction in *hORMDL3*^Myh11eGFP-cre^ mice.

### Does ORMDL3 increase intracellular Ca^2+^ levels by regulating Ca^2+^ channels at the ASM cell surface?

**Figure 6:**
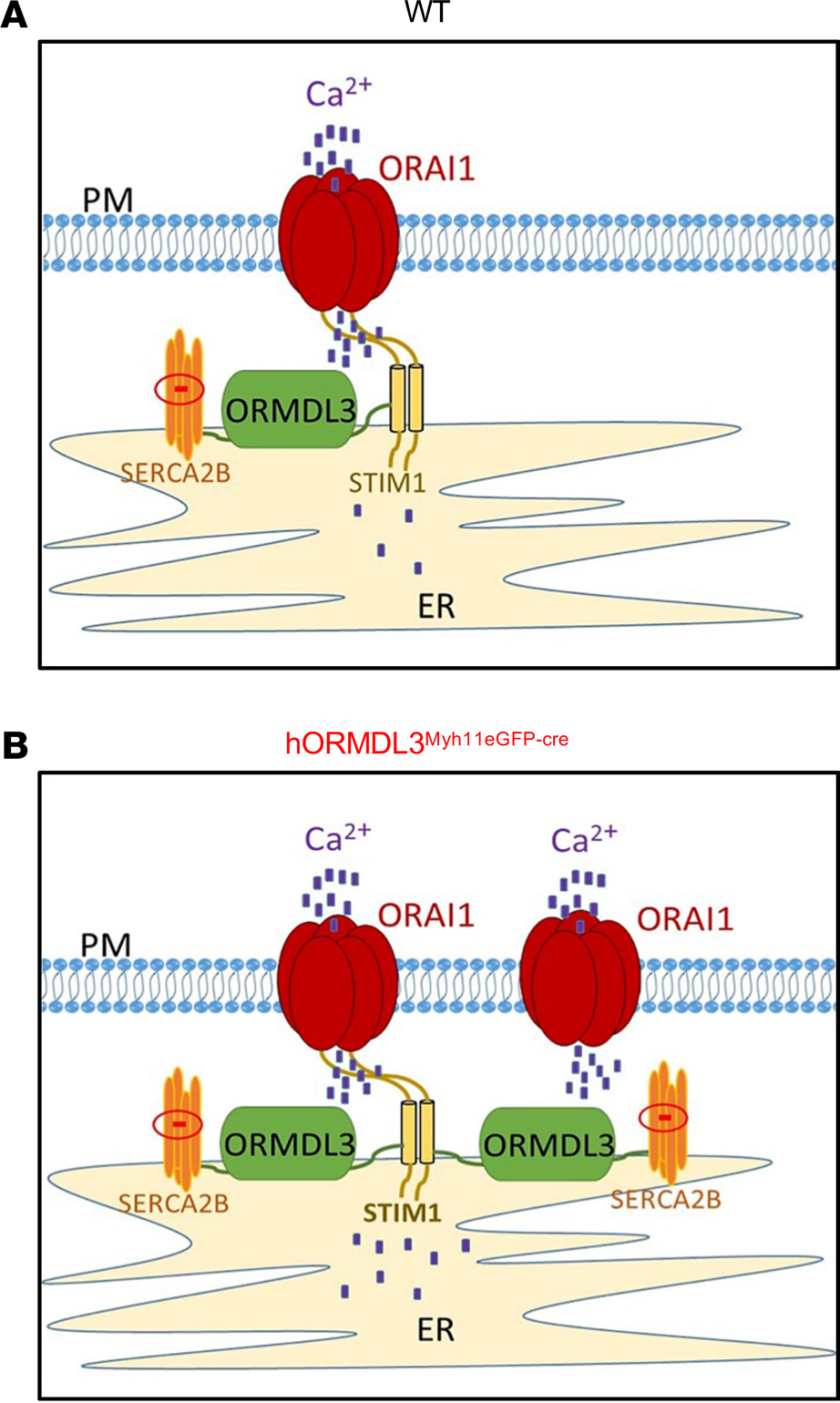
*ORMDL3* and regulation of Ca^2+^ in ASM. Overview of potential mechanisms involved in the regulation of baseline intracellular Ca^2+^ levels in ASM. (**A**) WT mouse ASM and (**B**) *hORMDL3*^Myh11eGFP-cre^ mouse ASM. (**B**) ASM from *hORMDL3*^Myh11eGFP-cre^ mice have higher baseline levels of intracellular Ca^2+^ (depicted as blue dots), more Orai1 channels (through which extracellular Ca^2+^ enters the cell) at the cell surface plasma membrane (PM), as well as more SERCA2b channels and more *ORMDL3* in the ER compared with (**A**) WT ASM. Levels of the intracellular Ca^2+^ sensor STIM1 are similar in ASM derived from *hORMDL3*^Myh11eGFP-cre^ and WT mice.

**Figure 7:**
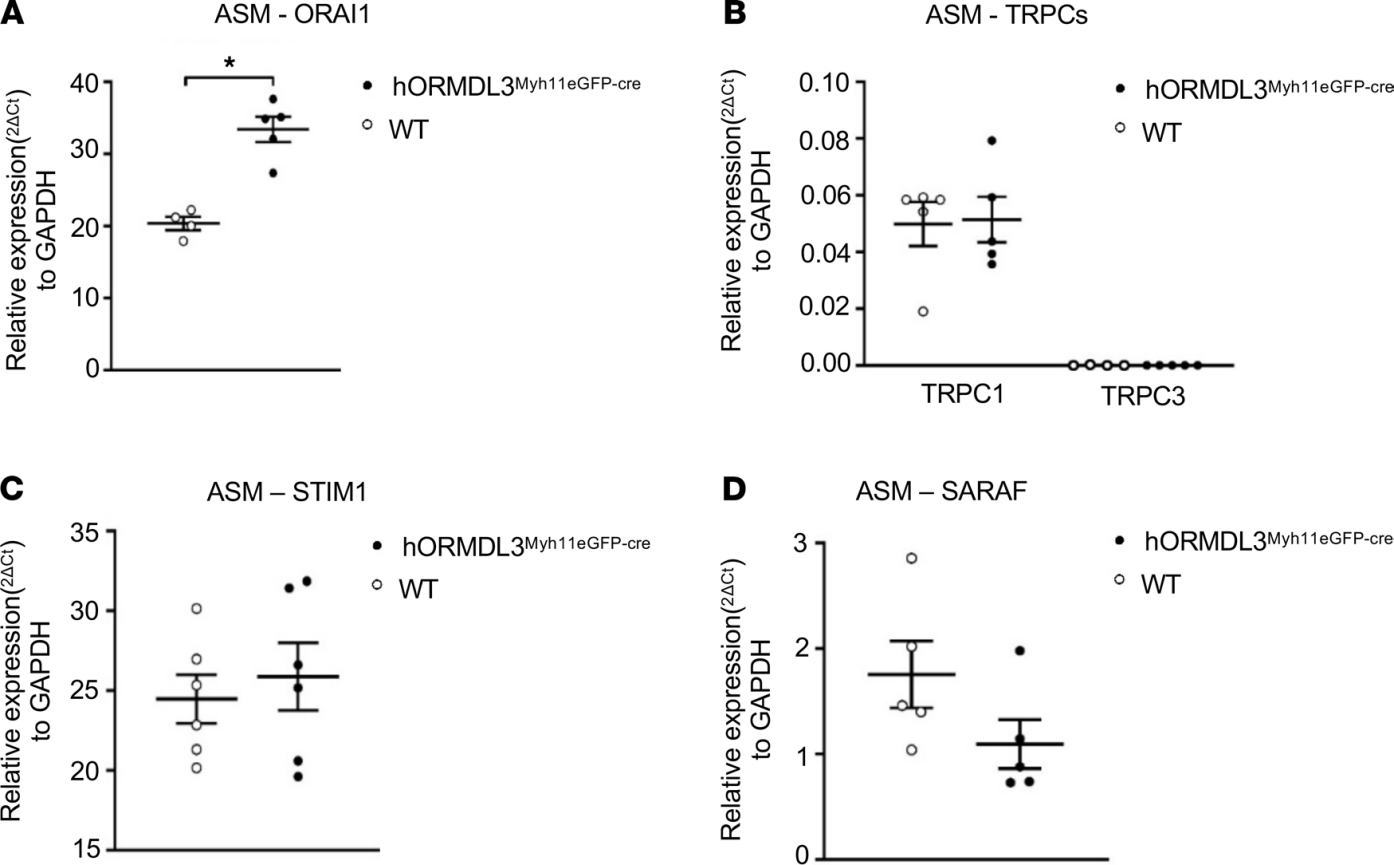
Effect of *ORMDL3* on ASM expression of mRNAs encoding calcium channels and calcium sensors. (**A**) ASM cell surface Ca^2+^ channel expression including (**A**) Orai1 and (**B**) TRPC1 and TRPC3 mRNA were assessed by RT-qPCR in WT and *hORMDL3*^Myh11eGFP-cre^ ASM (*n* = 4–6/group). (**C** and **D**) ER-associated and calcium-sensing STIM1 and SARAF mRNA levels were also assessed via RT-qPCR in WT and *hORMDL3*^Myh11eGFP-cre^ ASM (*n* = 4–6/group). *P < 0.05 compared with WT. *P* values are results of unpaired 2-tailed *t* tests.

The coordinated interactions of a number of proteins from the plasma and ER membranes control store-operated calcium entry (SOCE) to replenish internal Ca^2+^ stores and generate intracellular Ca^2+^ signals (19). As expression of *ORMDL3* in ASM increased intracellular Ca^2+^ levels, we investigated whether *ORMDL3* regulated pathways that could increase intracellular Ca^2+^ levels. Potential pathways linking the ER (where *ORMDL3* is localized) (2) to the cell surface plasma membrane to influence cell surface Ca^2+^ influx include effects of *ORMDL3* on cell surface calcium channels, which regulate cell surface Ca^2+^ influx (includes Orai1 channels, transient receptor potential channels, refs. 20, 21), and/or effects of *ORMDL3* on either stromal interaction molecule 1 (STIM1), an ER-resident Ca^2+^ sensor (22), or store-operated calcium entry associated regulatory factor (SARAF) (23), an ER-resident component of the SOCE, which serves as an important brake on SOCE (Figure 6). We therefore used qPCR to quantitate ASM levels of these cell surface Ca^2+^ channels (Orai1, transient receptor potential channels [TRPCs]) and the ER-resident Ca^2+^ sensors STIM1 and SARAF in *hORMDL3*^Myh11eGFP-cre^ and WT mice. These studies demonstrated that *ORMDL3* expression in ASM derived from *hORMDL3*^Myh11eGFP-cre^ mice had significantly increased levels of the Orai1 cell surface Ca^2+^ channel, which mediates Ca^2+^ influx into the cytoplasm (*P* < 0.05) (Figure 7A). In contrast, *ORMDL3* expression in ASM derived from *hORMDL3*^Myh11eGFP-cre^ mice did not regulate levels of TRPC cell surface Ca^2+^ channels (TPRC1, TPRC3) (Figure 7B) or levels of either STIM1 (Figure 7C) or SARAF (Figure 7D), which prevents cells from overfilling with Ca^2+^ (23). Cell surface Orai1 channels are components of 2 different SOCE channels involved in Ca^2+^ flux at the cell surface, namely calcium release activated calcium (CRAC) channels (composed of Orai1 subunits) (22), and SOC channels (composed of Orai1 subunits as well as TRPC subunits) (19). As *ORMDL3* influences only levels of Orai1, but not TRPC, it is more likely to affect levels of CRAC channels (composed of Orai1 subunits) as compared with SOC channels (composed of Orai1 and TRPC subunits). We also found no significant difference in the levels of the Orai1 regulators Septin (24) in ASM from *hORMDL3*^Myh11eGFP-cre^ and WT mice (Supplemental Figure 2B) to explain the increase in Orai1 channel levels induced by ORMDL3 (Figure 7A). In addition, we found no difference in levels of calcium release activated channel regulator 2A (CRACR2A, a cytosolic Ca^2+^ sensor that stabilizes CRAC channels) (25) in ASM from *hORMDL3*^Myh11eGFP-cre^ and WT mice (Supplemental Figure 2B).

### hORMDL3^Myh11eGFP-cre^ mice ASM express increased contractile genes (SERCA2b and SM22, but not Caldesmon or myosin light chain kinase 3).

**Figure 8:**
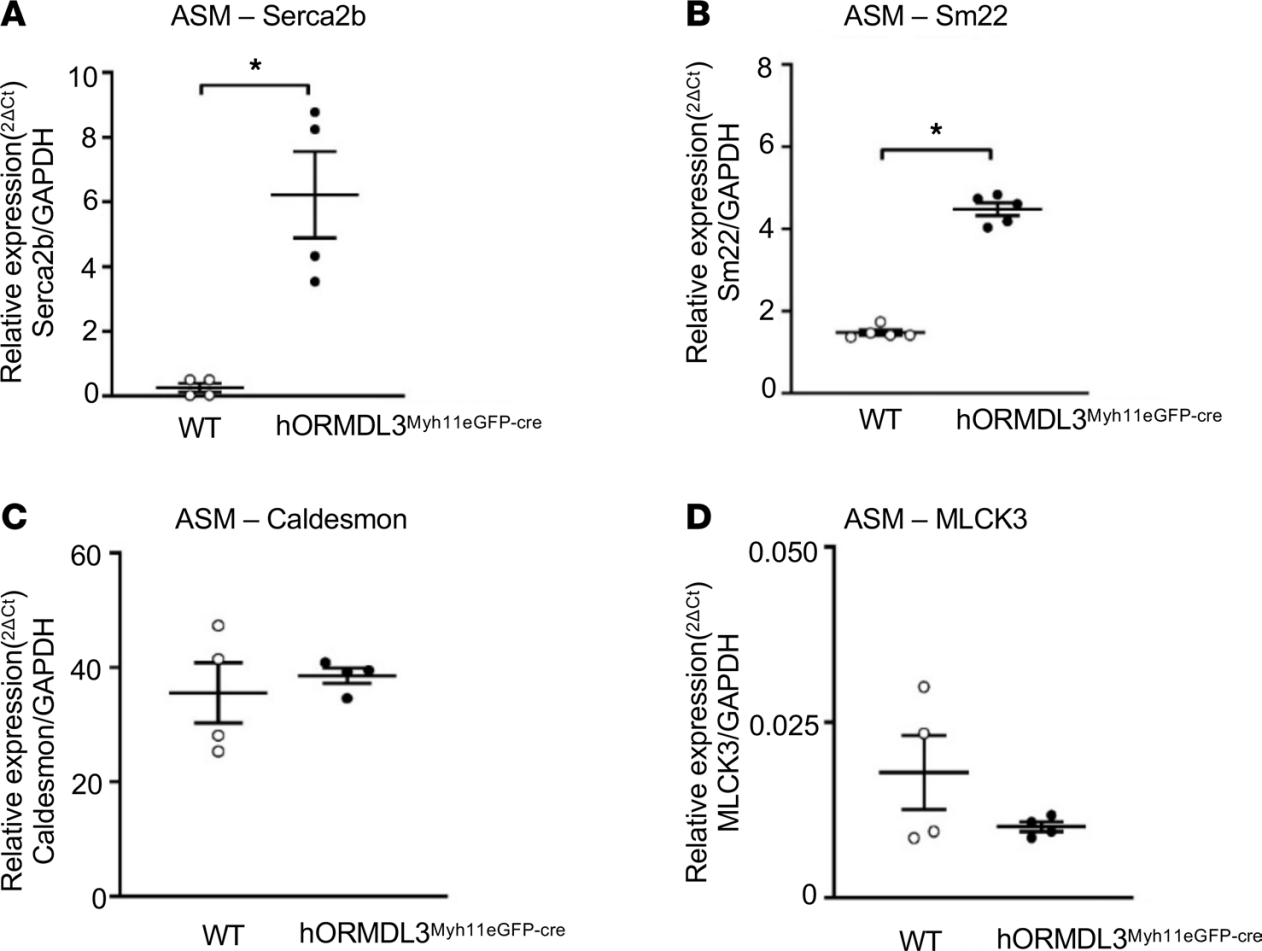
Effect of *ORMDL3* on ASM expression of contractile mRNA. (**A**) ASM cell expression of (**A**) SERCA2b, (**B**) Sm22, (**C**) Caldesmon, and (**D**) MLCK3 mRNA were assessed by RT-qPCR in WT and *hORMDL3*^Myh11eGFP-cre^ ASM (*n* = 4–5/group). *P < 0.05 compared with WT. *P* values are results of unpaired 2-tailed *t* tests.

As ASM derived from *hORMDL3*^smgfp-cre^ mice had increased contractility and increased intracellular Ca^2+^ levels, we examined whether *ORMDL3*-expressing ASM had markers associated with a contractile ASM phenotype, including SERCA2b, SM22, myosin light chain kinase 3 (MLCK3), and Caldesmon (26–29). Levels of expression of ASM contractile genes Serca2b (*P* < 0.001) (Figure 8A) and Sm22 mRNA (*P* < 0.05) (Figure 8B), but not Caldesmon (Figure 8C) or MLCK3 (Figure 8D) mRNA, were significantly higher in ASM cells from *hORMDL3*^Myh11eGFP-cre^ mice compared with WT mice. This suggests that *ORMDL3* expression in ASM can lead to altered gene expression of selected genes (SERCA2b, SM22) known to be involved in smooth muscle cell contraction (29, 30), a feature of asthma.

Previous studies have demonstrated that *ORMDL3* is localized to the ER (2) and that the ER plays an important role in regulating cytosolic calcium levels through SERCA pumps, which accumulate Ca^2+^ in the ER lumen (30). As our studies demonstrate that *ORMDL3* upregulated ASM levels of SERCA2b (Figure 8A), a Ca^2+^ pump localized to the sarcoplasmic endoplasmic reticulum in close proximity to *ORMDL3*, *ORMDL3* can through increased SERCA2b increase accumulation of Ca^2+^ in the ER lumen from the cytoplasm. However, as this *ORMDL3*-induced SERCA2b mediates movement of Ca^2+^ within 2 intracellular compartments (i.e., from the cytoplasm to the ER lumen), it should not increase overall intracellular Ca^2+^ levels, which we have noted in ASM cells from *hORMDL3*^Myh11eGFP-cre^ mice.

### Increased ASM (but not fibrosis or mucus) in airways of hORMDL3^Myh11eGFP-cre^ mice.

**Figure 9:**
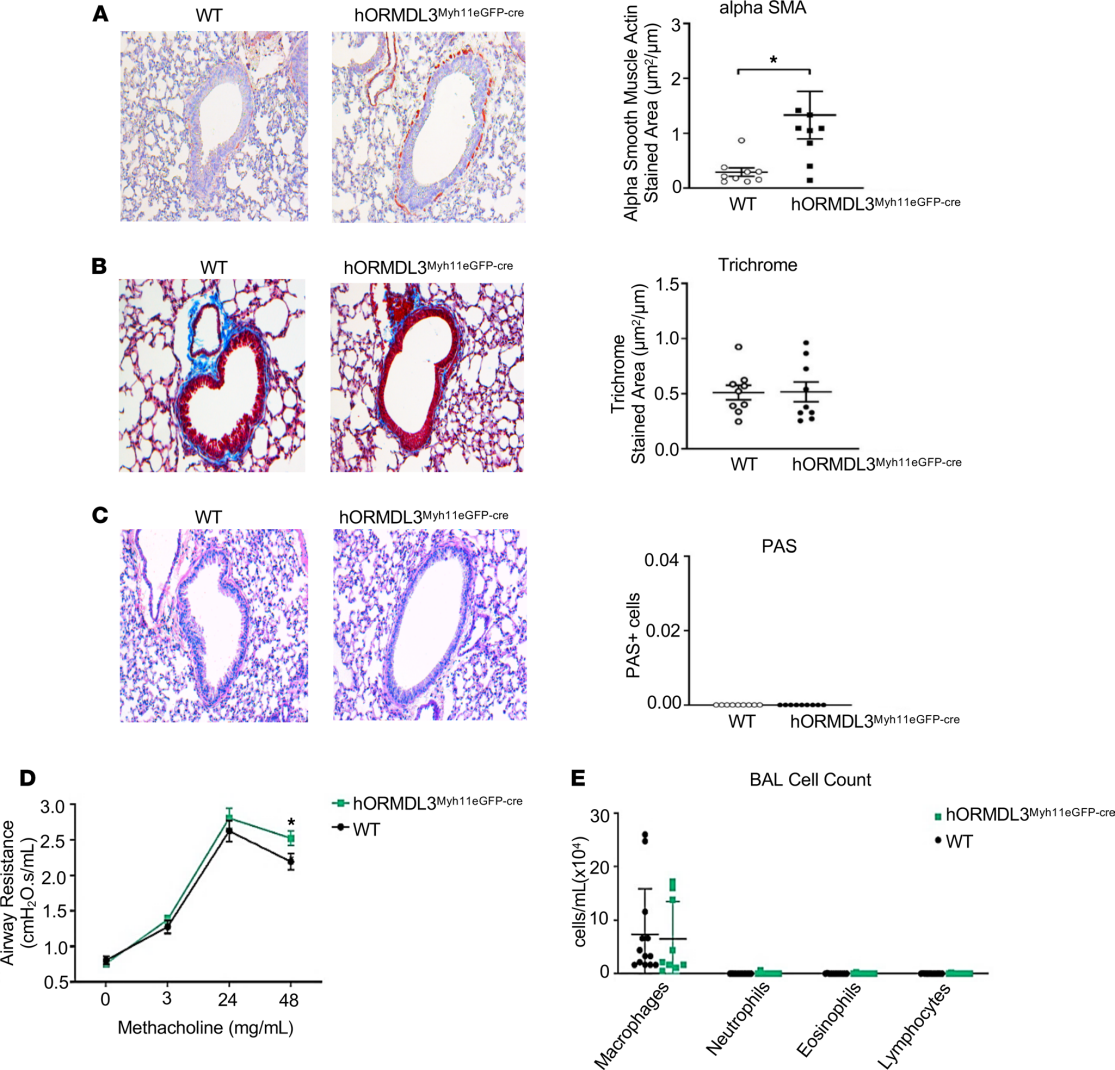
Effect of *ORMDL3* on airway remodeling, AHR, and airway inflammation in vivo. (**A**) WT and *hORMDL3*^Myh11eGFP-cre^ mice were sacrificed at 12 weeks of age in the absence of exposure to allergen. Lungs were used to quantitate by image analysis (**A**) ASM (alpha–smooth muscle actin–stained area) (*n* = 8–9 mice/group), (**B**) peribronchial fibrosis (trichrome-stained area) (*n* = 9 mice/group), and (**C**) mucus periodic acid–Schiff–positive epithelial cells (*n* = 8–9 mice/group). (**D**) Airway resistance following MCh challenge was assessed in intubated mice (*n* = 9 mice/group). (**E**) Airway inflammation was determined by quantitation of total BAL cells, macrophages, neutrophils, eosinophils, and lymphocytes in WT and *hORMDL3*^Myh11eGFP-cre^ mice (*n* = 9 mice/group). *P < 0.05 compared with WT. *P* values are results of unpaired 2-tailed *t* tests.

To determine whether mice expressing increased ORMDL3 in ASM selectively had increased ASM in vivo, we quantitated levels of ASM in *hORMDL3*^Myh11eGFP-cre^ and WT mice aged 12 weeks that had not been exposed to any environmental stimulus, such as allergen challenge. Previous studies had demonstrated that at 12-week-old mice universally expressing increased *ORMDL3* in all cells spontaneously developed increased ASM, increased peribronchial fibrosis, and increased mucus (8). Selective expression of *ORMDL3* in ASM in *hORMDL3*^Myh11eGFP-cre^ mice resulted in spontaneous significantly increased peribronchial ASM assessed by image analysis quantitation of the area of ASM immunostaining with an antibody against alpha–smooth muscle actin (*P* < 0.05) (Figure 9A). In contrast to universal *ORMDL3*-Tg mice, which also exhibited increased peribronchial fibrosis and increased mucus (8), selective expression of *ORMDL3* in ASM in *hORMDL3*^Myh11eGFP-cre^ mice did not show evidence of increased peribronchial fibrosis (Figure 9B) or increased mucus (Figure 9C).

### AHR to methacholine in hORMDL3^Myh11eGFP-cre^ mice.

*hORMDL3*^Myh11eGFP-cre^ mice not exposed to allergen had a small but statistically significant spontaneous increase in airway responsiveness to methacholine (MCh) 48 mg/mL compared with WT mice at 12 weeks of age (*P* < 0.05) (Figure 9D). *hORMDL3*^Myh11eGFP-cre^ mice challenged with house dust mite (HDM) had a significant increase in AHR compared with nonchallenged *hORMDL3*^Myh11eGFP-cre^ mice (*P* = 0.0061) (Supplemental Figure 3A). However, there was no increase in AHR in *hORMDL3*^Myh11eGFP-cre^ mice challenged with HDM compared with WT mice challenged with HDM (Supplemental Figure 3A). The small but statistically significant spontaneous increase in airway responsiveness to MCh noted in *hORMDL3*^Myh11eGFP-cre^ mice in the initial experiment (Figure 9D) was again noted in the nonchallenged control *hORMDL3*^Myh11eGFP-cre^ mice group in the acute HDM experiment (*P* = 0.0253, nonchallenged control *hORMDL3*^Myh11eGFP-cre^ mice vs. nonchallenged WT) (Supplemental Figure 3A).

### Quantitation of airway inflammation in BAL in hORMDL3^Myh11eGFP-cre^ mice.

*hORMDL3*^Myh11eGFP-cre^ mice did not develop any spontaneous evidence of airway inflammation at 12 weeks of age (the time point they had spontaneous increased ASM) (Figure 9A) as assessed by quantitation of total cells, macrophages, lymphocytes, eosinophils, and neutrophils in BAL (Figure 9E). Levels of BAL eosinophils were similar in acute HDM–challenged *hORMDL3*^Myh11eGFP-cre^ mice and HDM-challenged WT mice (Supplemental Figure 3B).

## Discussion

In this study we provide potentially novel evidence that *ORMDL3* expression selectively in ASM can induce increased ASM hypertrophy, proliferation, and contractility, which together may help explain the epidemiologic linkage of chromosome 17q12-21 to the development of childhood onset asthma (1) (where ASM can express *ORMDL3* in the lower airway) but not to the development of allergic rhinitis (7) (where there are no ASM cells in the nasal mucosa). In addition to demonstrating that ORMDL3 expression in ASM induced functional changes (i.e., increased ASM hypertrophy, proliferation, and contractility), we identified potentially novel pathways through which *ORMDL3* may contribute to these ASM changes characteristic of asthma. These pathways we identified to be *ORMDL3* regulated in ASM include (a) TPM1 and TPM4 mediated ASM hyperplasia, (b) *ORMDL3* regulated Ca^2+^ signaling (increased levels of intracellular Ca^2+^, increased cell surface membrane Orai1 Ca^2+^ channels, which mediate influx of Ca^2+^ into the cytoplasm), and (c) *ORMDL3* upregulated expression of ASM contractile genes (Serca2b and Sm22). In addition, the potential importance of *ORMDL3*-induced increases in Serca2b in ASM to *ORMDL3*-induced ASM hypertrophy is suggested from studies of the heart, in which mice expressing increased levels of Serca2b exhibited cardiac muscle hypertrophy and increased contractility (30, 31). Interestingly, in our study mice selectively expressing human *ORMDL3* in ASM developed increased expression of Serca2b, ASM hypertrophy, and increased contractility, suggesting a potential link between Serca2b and ASM hypertrophy and contractility not only in cardiac muscle (30, 31) but also in ASM, in which levels of *ORMDL3* are increased. Thus, childhood asthmatics with SNPs on chromosome 17q12-21 linked to increased *ORMDL3* expression may have increased amounts of peribronchial smooth muscle due to ASM hypertrophy and hyperplasia. In addition, it is also likely that *ORMDL3* expression in cells other than ASM, such as CD4^+^ cells, results in enhanced Th2 responses (4), further increasing AHR through effects of Th2 cytokines such as IL-13 on ASM (32) to increase AHR.

The increased thickness of the ASM layer is a characteristic feature of asthma and is due to both ASM hypertrophy (10–12) and hyperplasia (10, 13, 14), without a change in the proportion of extracellular matrix, all of which are related to asthma severity (33). In this study we have demonstrated that *ORMDL3* expression in ASM induces both ASM hypertrophy and hyperplasia, without a change in expression of extracellular matrix proteins, recapitulating many of the findings noted in asthmatics. While many studies in mice use tracheal smooth muscle, an advantage of this study is that we used mouse bronchial smooth muscle (of greater relevance to asthma than tracheal smooth muscle), which expressed copy numbers of human *ORMDL3* similar to that noted in ASM in human patients with asthma. Evidence that *ORMDL3* regulated ASM hypertrophy was noted in studies using FACS, protein/DNA ratio, and microscopic image analysis. While many cytokines/mediators or mechanical stretch signals arising extrinsic to ASM have been identified to modulate ASM hypertrophy and hyperplasia (17, 34, 35), this study makes the likely novel observation of the importance of intrinsic expression in ASM of a gene (i.e., *ORMDL3*) on chromosome 17q12-21 to the development of ASM hypertrophy and hyperplasia. Thus, children with SNPs linked to chromosome 17q12-21 and increased expression of *ORMDL3* in ASM may develop a childhood onset increased thickness of the ASM layer, predisposing to the development of childhood onset asthma.

This study also demonstrated the importance of TPM (16) to *ORMDL3*-induced ASM proliferation, which can manifest as ASM hyperplasia in the airway in asthma. TPM has not previously been linked to *ORMDL3* or to ASM proliferation, underscoring the novelty of this finding. In addition, only 2 of the 4 TPM genes (i.e., TPM1 and TPM4 but not TPM2 or TPM3) were regulated by *ORMDL3*. TPM is a coiled-coil dimer protein that lies end to end in the actin groove and plays an important role in regulating muscle contraction (16). Thus, *ORMDL3* upregulation of TPM1 and TPM4 could regulate not only ASM proliferation but also ASM contractility. ASM contraction results from the interaction of myosin with F-actin, which is controlled by calcium ions via regulatory proteins including TPM associated with F-actin (16). Therefore, the ability of *ORMDL3* to regulate TPM1, TPM4, and Ca^2+^ may have important implications for ASM contractility. Support for the importance of TPMs to muscle contraction is also derived from studies of cardiac muscle in which *TPM1* mutations have been associated with systolic hypercontractility in hypertrophic obstructive cardiomyopathy (36).

In addition, we identified that *ORMDL3* regulated increased expression of ASM contractile genes (Serca2b and Sm22). Serca2b is an intracellular ATP-driven Ca^2+^ pump that transports Ca^2+^ from the cytoplasm into the sarco-endoplasmic reticulum (26, 37). Based on studies of increased expression of Serca2b in cardiac muscle (30, 31), increased expression of Serca2b in ASM may be the downstream pathway by which *ORMDL3* influences ASM Ca^2+^ flux, contractility, and hypertrophy. For example, studies of mice expressing increased levels of Serca2b in the heart had increased cardiac contractility with increased Ca^2+^ flux in cardiomyocytes and increased cardiac muscle hypertrophy (30, 31). Interestingly, knockdown of SERCA2b in ASM reduces ASM contractility in vitro (6), defining similarities in SERCA2b function as it relates to contractility in both the heart (30, 31) and in ASM (6). While a study has reported that decreased expression of SERCA in ASM from asthmatics (who were not genotyped for chromosome 17q12-21 SNPs or enrolled based on history of childhood onset asthma) contributes to airway remodeling (38), we hypothesize that genotyping childhood asthmatics for chromosome 17q12-21 SNPs may identify a subset of asthmatics with increased *ORMDL3* and SERCA2b expression. In addition to *ORMDL3* regulating SERCA2b, we also noted that *ORMDL3* regulated increased levels of SM22a (transgelin), an actin filament–associated protein in smooth muscle (29). Studies of endobronchial biopsies from asthmatics have shown by qPCR and immunohistochemistry that levels of SM22 are increased in ASM in asthma compared with nonasthmatics (39). Although SM22 is highly expressed in ASM and binds to filaments of actin in smooth muscle cells in vitro and in vivo (29), its function in ASM requires further study in asthma using allergen or viral challenge models because it is not essential for constitutive smooth muscle development as demonstrated in Sm22-KO mice (40). Thus, *ORMDL3* expression in ASM is associated with increased expression of selected ASM contractile proteins (Serca2b, SM22a, but not MLCK3) and increased contractility.

In this study we also made the potentially novel observation that ASM from *hORMDL3*^Myh11eGFP-cre^ mice has increased basal total intracellular Ca^2+^ levels, which are associated with increased basal ASM contractility. When cytosolic Ca^2+^ levels are increased, the combination of Ca^2+^ and calmodulin activate myosin light chain (MLC) kinase, which phosphorylates MLC, leading to enhanced ASM contraction (17, 41). In previous studies we have demonstrated that lung slices from universal *ORMDL3*-Tg mice exhibit increased Ca^2+^ oscillations when stimulated with acetylcholine (6), but prior studies have not examined effects of selective *ORMDL3* expression in ASM on total intracellular Ca^2+^ levels or *ORMDL3* effects on Ca^2+^-replenishing pathways in ASM (Serca2b, STIM1, Orai1, or regulators of Orai1). Ca^2+^ oscillations occur as the Ca^2+^ is taken up into sarcoplasmic reticulum (SR) stores via SERCA. ASM Ca^2+^ stores are then replenished via SERCA and by the oligomerization of the SR-localized STIM1 (a Ca^2+^ sensor), which then associates with Orai1 in the plasma membrane, causing it to form channels, which mediate SOCE (42). In this study, we advance our knowledge of how *ORMDL3* regulates intracellular Ca^2+^ in ASM to demonstrate that *ORMDL3* increases total intracellular Ca^2+^ levels and that this is associated with increased Serca2b (which is able to replenish SR Ca^2+^ stores), as well as increased Orai1, which mediates cell surface SOCE. Thus, increased total intracellular Ca^2+^ in ASM may be mediated by *ORMDL3* located in the ER regulating a cell surface filling mechanism for Ca^2+^ entry mediated by Orai1. This *ORMDL3*-regulated Orai1 cell surface filling mechanism for Ca^2+^ entry would be controlled by the filling state of intracellular Ca^2+^ and by SOCE, which has been shown to depend on the cell surface transmembrane protein Orai1 and its interaction with STIM1, an ER Ca^2+^ sensor (43). Ca^2+^ influx due to low ER stores gives rise to the Ca^2+^ released activated Ca^2+^ current (I*_CRAC_*), which then activates Orai1 to open in order to replenish cytosolic Ca^2+^ concentration. Interestingly, allergen-challenged mice have increased levels of Orai1 in ASM (44).

In contrast to our study demonstrating that *ORMDL3* upregulates Ca^2+^ levels and Orai1 levels in primary mouse ASM cells, studies in a human Jurkat T cell leukemia cell line transfected with *ORMDL3* have shown that *ORMDL3* negatively modulates SOCE by inhibiting Ca^2+^ influx and inhibits STIM1 in Jurkat cells (45, 46). It should be noted that in our studies the *ORMDL3* mRNA copy number in mouse ASM we studied, which was derived from *hORMDL3*^Myh11eGFP-cre^ mice, was similar to the *hORMDL3* copy number detected in ASM from human patients with asthma. The differences in the results from the 2 studies may relate to the difference in cell types studied (primary mouse ASM cells vs. leukemic T cell line), methods of generating increased *ORMDL3* (transgenic mice vs. transfection), methods used (intracellular Ca^2+^ dye Fura2 vs. Fluo-4, cresolphthalein), outcomes studied (PCR for Stim1), and/or other factors.

While previous studies of universal *ORMDL3*-expressing transgenic mice (8) and their precision-cut lung slices (6) have demonstrated that universal *ORMDL3* expression in all cells results in spontaneous increased AHR to MCh, this study markedly extends these observations to demonstrate that selective *ORMDL3* expression in ASM only (rather than in any other lung, immune, or inflammatory cell in universal *ORMDL3*-Tg mice) (6, 8) is inducing increased ASM contractility as well as ASM hypertrophy, which were not studied in universal *ORMDL3*-Tg mice. Studies of *hORMDL3*^Myh11eGFP-cre^ mice also demonstrated that they had spontaneous increased levels of ASM but not spontaneous increased amounts of peribronchial fibrosis or mucus, which were noted in universal *ORMDL3*-Tg mice (8). Thus, cells expressing *ORMDL3* other than ASM are likely to contribute to spontaneous peribronchial fibrosis and mucus. In addition, this study demonstrated that selective *ORMDL3* expression in ASM regulates likely novel pathways not studied in universal *ORMDL3*-Tg mice, including *ORMDL3* regulation of ASM hypertrophy, TPM proteins involved in ASM hyperplasia, increased expression of ASM contractile genes (Serca2b and Sm22), increased Orai1 Ca^2+^ channels, and increased intracellular Ca^2+^ levels, providing additional pathways through which *ORMDL3* may regulate ASM contractility, which may contribute to the development of AHR in childhood onset asthma, which is highly linked to *ORMDL3* on chromosome 17q12-21.

Our studies demonstrated that nonchallenged *hORMDL3*^Myh11eGFP-cre^ mice had a modest, but reproducible, statistically significant spontaneous increase in AHR compared with nonchallenged WT mice in 2 different experiments. However, there was no increase in AHR in *hORMDL3*^Myh11eGFP-cre^ mice challenged with HDM compared with WT mice challenged with HDM. This result may reflect that there is only a small but reproducible increase in AHR in nonchallenged *hORMDL3*^Myh11eGFP-cre^ mice compared with nonchallenged WT mice. In addition, the allergen stimulus we used, HDM, induces a robust increase in AHR, which may mask the contribution to AHR provided by *ORMDL3* in ASM. It is also likely that *ORMDL3* expression in cells other than ASM, such as CD4^+^ cells, results in enhanced Th2 responses (4), further increasing AHR through effects of Th2 cytokines such as IL-13 on ASM (32) to increase AHR. In support of this effect of *ORMDL3* expressed in CD4^+^ cells on AHR, prior studies of universal *ORMDL3*-Tg mice (8) demonstrated a greater increase in AHR compared with our studies of selective expression of *ORMDL3* in ASM in *hORMDL3*^Myh11eGFP-cre^ mice, suggesting that the combination of *ORMDL3* expression in ASM and other cell types (possibly CD4^+^ cells) is needed for significant enhancement in AHR. At present no studies have reported the effect of the selective expression of increased *hORMDL3* in mouse CD4^+^ cells on AHR.

In this study we utilized a mouse model in which we expressed increased levels of the human *ORMDL3* gene, as studies of human SNPs linked to *ORMDL3* in asthma demonstrate increased levels of *ORMDL3* (47). *ORMDL3* exhibits 96% identity between mouse and human (48), suggesting that mouse *Ormdl3* and human *ORMDL3* have similar functions. Although we expressed human *ORMDL3* in the presence of the endogenous mouse *Ormdl3* in *hORMDL3*^Myh11eGFP-cre^ mice, we do not think the endogenous mouse *Ormdl3* influenced our results, as the control WT mice, like the *hORMDL3*^Myh11eGFP-cre^ mice, expressed similar levels of the endogenous mouse *Ormdl3*. Thus, the only difference in *ORMDL3* between the *hORMDL3*^Myh11eGFP-cre^ mice and WT mice was the level of human *ORMDL3*, which was the focus of this study.

In summary, our studies demonstrate that *ORMDL3* expression selectively in ASM can induce spontaneous (in the absence of allergen or other environmental trigger) increased ASM hypertrophy, ASM proliferation, and contractility, which together could contribute to the development of AHR and childhood onset asthma. In addition to demonstrating that *ORMDL3* expression in ASM induced functional changes, we identified potentially novel pathways regulated by *ORMDL3* in ASM, including TPM1 and TPM 4 mediated ASM hyperplasia, and *ORMDL3* induced increased ASM contractility to histamine in vitro. These *ORMDL3*-induced pathways in ASM were associated with increased levels of intracellular Ca^2+^, increased cell surface membrane Orai1 Ca^2+^ channels — which mediate influx of Ca^2+^ into the cytoplasm — and increased expression of ASM contractile genes, including Serca2b and Sm22. These *ORMDL3*-induced changes in ASM may contribute to the development of AHR in childhood onset asthma, which is highly linked to *ORMDL3* on chromosome 17q12-21.

## Methods

### RFP-Stop^fl^*hORMDL3*-Tg mice

The generation of *hORMDL3*-Tg floxed mice (RFP-Stop^fl^*hORMDL3*-Tg) on a C57BL/6 background in this laboratory has been previously described (8).

### Myh11-creEGFP mice

Myh11-creEGFP mice (smooth muscle–specific cre) on a C57BL/6 background were acquired from The Jackson Laboratory (9).

### *hORMDL3*^Myh11eGFP-cre^ mouse ASM cells for in vitro study

Mouse ASM cells were obtained from the bronchial airways dissected free of lung tissue of *hORMDL3*^Myh11eGFP-cre^ and WT mice (Figure 1, A–C). Mice were euthanized and the tracheobronchial tree free of lung tissue was isolated up to the third generation by dissecting and removing the surrounding lung parenchyma and vasculature from major bronchi under a dissecting microscope (Figure 1, B and C) (Leica DMLS, Leica Microsystems). The trachea was detached from the bronchial tree, and only the bronchial tree was digested in a cocktail of collagenases (Roche Diagnostics) for 30–60 minutes at 37°C to generate a single-cell suspension of bronchial cells. Cells were then cultured in mouse smooth muscle cell media (Cell Biologics) for 3 to 4 weeks at which time 90% of the cells were ASM cells as defined by expression of ASM genes (alpha–smooth muscle actin), expression of EGFP, and morphology.

### Generation of *hORMDL3*^Myh11eGFP-cre^ Tg mice

To generate *hORMDL3*^Myh11eGFP-cre^ mice, we crossed RFP-Stop^fl^*hORMDL3*-Tg mice (which have a floxed transcriptional stop codon positioned in front of the *hORMDL3* transgene preventing *hORMDL3* transcription) with a smooth muscle–specific cre mouse (Myh11-creEGFP) (cre expressed selectively in smooth muscle cells under the smooth muscle myosin heavy chain Myh11 promoter tagged with EGFP) (Figure 2A). Crossing the RFP-Stop^fl^*hORMDL3*-Tg mice with Myh11-creEGFP mice results in smooth muscle–expressed cre excising the floxed transcriptional stop codon positioned in front of the *hORMDL3* transgene, which then allows *hORMDL3* expression in smooth muscle cells. *hORMDL3*^Myh11eGFP-cre^ and littermate (referred to as WT) mice were genotyped by PCR as previously described (8) to detect (a) *hORMDL3*: F1, 5′-GCAACGTGCTGGTTATTGTG-3′; F2, 5′-CCCCCTGAACCTGAAACATA-3′; R, 5′-TACAGCACGATGGGTGTGAT-3′; (b) Myh11Cre: F, GCGGTCTGGCAGTAAAAACTATC; R, GTGAAACAGCATTGCTGTCACTT; and (c) EGFP: F, AAGTTCATCTGCACCACCG; R, TCCTTGAAGAAGATGGTGCG.

The expected sizes of *hORMDL3*, Myh11Cre, and EGFP PCR products are 479 bp, 100 bp, and 173 bp, respectively (Figure 2B).

### Human ASM cells

Postmortem human lungs from asthmatics were procured by the Arkansas Regional Organ Recovery Agency and by the National Disease Research Interchange and delivered to the Lung Cell Biology Laboratory at the Arkansas Children’s Research Institute and processed for studies of the expression of *ORMDL3* mRNA copy number in ASM at UC San Diego as described (49). To obtain human ASM from postmortem asthma lungs, surrounding lung tissue was removed from bronchial airways, which were cut into small pieces (4–5 strips per airway) and transferred to a 6-well tissue culture dish for ASM attachment and outgrowth. After 7 days in culture, ASM outgrowth was present and the bronchial strips were removed. Human ASM cells were then maintained with smooth muscle cell (SMC) media (ScienCell Research Laboratories) for up to 5 passages at which time more than 90% of the cells were ASM cells as defined by expression of ASM genes (alpha–smooth muscle actin) and morphology.

### *hORMDL3* copy number in mouse *hORMDL3*^Myh11eGFP-cre^ and human ASM cells

Mouse ASM cells were derived from *hORMDL3*^Myh11eGFP-cre^ mice, and human ASM cells were derived from postmortem asthma lungs (49) as described above. *hORMDL3* mRNA copy number values were quantitated in mouse and human ASM by using a dPCR method as previously described in this lab (50). In brief, total RNA was extracted from WT and *hORMDL3*^Myh11eGFP-cre^ mouse ASM cells, as well as from human ASM cells, with RNA-STAT-60 (Tel-Test) and reverse-transcribed with the Oligo-dT and SuperScript II Kit (Life Technologies, Thermo Fisher Scientific). *hORMDL3* mRNA copy number values were quantitated by using a dPCR method, in which the reaction mixture contained 500 ng of target cDNA, Supermix (Bio-Rad Laboratories), as well as *hORMDL3*-specific primers and probes (Applied Biosystems, Thermo Fisher Scientific). The reaction mixture was loaded into the sample well of a disposable droplet generator cartridge (Bio-Rad Laboratories). After droplet generation, the mixture was transferred into a 96-well PCR plate and then amplified on the Bio-Rad T100 Thermal Cycler. The plate was analyzed with a QX200 Droplet Reader, and the dPCR data were processed by using QuantaSoft software (Bio-Rad Laboratories). Results are expressed as *hORMDL3* mRNA copy number normalized to mouse or human HPRT.

### Selective expression of *hORMDL3* transgene in ASM in *hORMDL3*^Myh11eGFP-cre^ mice

To determine smooth muscle–specific expression of the *hORMDL3* transgene in *hORMDL3*^Myh11eGFP-cre^ mice, we obtained purified populations of ASM, bronchial epithelial cells, and BAL macrophages. Bronchial epithelial cells were obtained using a bronchial brushing technique previously described in this laboratory (51). BAL macrophages were obtained by inserting a 20-gauge catheter into the trachea and lavaging the lungs with 1 mL of PBS/EDTA, which was repeated on 5 occasions (52). Expression of the *hORMDL3* transgene in ASM, bronchial epithelial cells, and BAL macrophages from *hORMDL3*^Myh11eGFP-cre^ and WT mice was assessed via RT-qPCR. We also used RT-qPCR to quantitate endogenous levels of mouse *Ormdl3* in ASM as described (2).

### ASM cell size in *hORMDL3*^Myh11eGFP-cre^ mice

We used 3 methods (FACS, image analysis, and protein/DNA ratio) to quantitate ASM cell size.

#### FACS.

ASM cell size was compared in *hORMDL3*^Myh11eGFP-cre^ and WT ASM cells by FACS analysis using measurements of forward scatter after gating on live cells with a Novocyte cytometer (Acea Biosciences, Inc).

#### Image analysis.

To compare ASM cell area (as an index of hypertrophy) in ASM derived from *hORMDL3*^Myh11eGFP-cre^ with that of WT mice, ASM cells were cultured on cell culture chamber slides for 24 hours. Adapting image analysis methods previously used for quantitation of cardiac SMC size (53), ASM cells were fixed with paraformaldehyde and stained with phalloidin-488 (Thermo Fisher Scientific), which stains the F-actin filament of ASM (53). Images of individual ASM cells were captured in 10 random fields, and ASM cell area was measured using the ImageJ software (NIH). Results are expressed as ASM cell area in square micrometers.

#### Protein/DNA ratio.

We also compared protein/DNA ratio in ASM derived from *hORMDL3*^Myh11eGFP-cre^ with that of WT mice as an index of ASM hypertrophy as described (54). Protein/DNA ratio has been used as an indicator of cellular hypertrophy, as with cell size increase (i.e., hypertrophy), more protein synthesis occurs (relative to DNA synthesis) to meet the increased architectural and functional demands of the growing cell (55). To determine ASM hypertrophy, total protein to DNA content was assessed in WT and *hORMDL3*^Myh11eGFP-cre^ ASM cells seeded at a density of 2 × 10^5^ cells/well for 2 days. Protein and DNA were then isolated using Qiagen AllPrep kit, and total protein and DNA content was quantified using a NanoDrop Spectrophotometer (Thermo Fisher Scientific).

### ASM hyperplasia in *hORMDL3*^Myh11eGFP-cre^ mice

To compare ASM proliferation (as an index of ASM hyperplasia) in vitro in ASM derived from *hORMDL3*^Myh11eGFP-cre^ and WT mice, we measured BrdU incorporation in ASM cells using a BrdU cell proliferation ELISA (Exalpha Biologicals Inc). In brief, ASM cells were seeded at 2 × 10^4^ cells/well overnight in a 96-well plate. BrdU was incubated for 18 hours with ASM cells cultured in the presence or absence of 10% FBS (FBS stimulates ASM proliferation) (56). BrdU incorporation was measured by ELISA according to the manufacturer’s instructions.

### *hORMDL3*^Myh11eGFP-cre^ mouse expression of ASM cell synthetic, contractile, and Ca^2+^ flux genes

We utilized RT-qPCR to quantitate levels of expression of synthetic genes including tropomysins (TPM1, TPM2, TPM3, TPM4) (16), Myh10 (57), and vimentin (58); contractile genes including Serca2b (26), MLCK3 (27), Caldesmon (28), and SM22 (29); Ca^2+^ flux-regulating genes including STIM1 (22), Orai1 (20), TRPC1 and TRPC3 (21), and SARAF (23); regulators of Orai1 channels including CRACR2A (25) and SEPTIN (24); as well as extracellular matrix genes expressed by ASM (59) including laminin, perlecan, collagen, and fibronectin in ASM derived from *hORMDL3*^Myh11eGFP-cre^ and WT mice. RT-qPCR was performed as previously described in this laboratory (8). In brief, total RNA was extracted with RNA-STAT-60 (Tel-Test) and reverse-transcribed with Clontech cDNA Synthesis Kit. RT-qPCR was performed with TaqMan PCR Master Mix, and primers were obtained from Life Technologies (Thermo Fisher Scientific). The relative amounts of transcripts were normalized to those of housekeeping gene (GAPDH) mRNA and compared between the different genes by the double delta Ct (ΔΔ) cycle threshold method as previously described in this laboratory (8).

### Effect of siRNA knockdown of TPM1 or TPM4 in ASM on proliferation and cell size

Mouse ASM cultures were grown in 6-well plates using SMC media with smooth muscle growth supplement (Cell Biologics). ASM cultures were transfected with 20 nM TPM1 siRNA, TPM4 siRNA, or control siRNA by using transfection reagents siTran1.0 (Origene) and Opti-MEM (Thermo Fisher Scientific) according to the manufacturers’ instructions. The transfected ASM cells were used 24 hours after the transfection in all experiments including quantitation of ASM proliferation, ASM cell size, and expression of TPM1 and TPM4 mRNA. Detection of expression of TPM1 and TPM4 mRNA by RT-qPCR was performed with TaqMan PCR Master Mix and mouse TPM1 or TPM4 primers (Life Technologies, Thermo Fisher Scientific). The relative amounts of transcripts were normalized to those of housekeeping gene (GAPDH) mRNA and compared between control siRNA–transfected samples and TPM1 siRNA– or TPM4 siRNA–transfected samples by the ΔΔ cycle threshold method as previously described in this laboratory (2, 8).

### *hORMDL3*^Myh11eGFP-cre^ mouse ASM cell contraction assay in vitro

Mouse ASM (passage 3) from *hORMDL3*^Myh11eGFP-cre^ and WT mice were used in an in vitro smooth muscle gel contraction assay previously described in this laboratory (6). In brief, ASM cells were seeded at a density of 2 × 10^5^ cells/well in collagen gels free of LPS (Advanced BioMatrix). After a 3-day incubation in collagen gels containing smooth muscle complete media (ScienCell Research Laboratories) containing 1.8 mM Ca^2+^ or similar media containing no calcium, *hORMDL3*^Myh11eGFP-cre^ or WT ASM cells were cultured in the presence or absence of an agonist (100 μM histamine) or media control for 0, 15, 30, 45, and 60 minutes. With agonist-induced ASM contraction, the area of the gel decreases significantly (6). The area of the gels was quantitated by using a Bio-Rad ImageDR transilluminator and Versadoc scanner (Bio-Rad Laboratories) with an accompanying image capture and analysis program to generate area in square millimeters. Results are expressed as the percentage of contracted gel area divided by the area at time 0 minutes.

### Levels of total intracellular Ca^2+^ in ASM in *hORMDL3*^Myh11eGFP-cre^ mice

Levels of total intracellular Ca^2+^ were measured in isolated ASM cells from WT and *hORMDL3*^Myh11eGFP-cre^ mice. Briefly, ASM cells were seeded at 2 × 10^5^ cells/well overnight in a 96-well plate with smooth muscle complete media (ScienCell Research Laboratories) containing 1.8 mM Ca^2+^. ASM cells were then collected and homogenized, and total calcium concentration was measured using a chromogenic assay. In this assay a complex is formed between calcium ions and 0-cresolphthalein and measured at optical density of 575 nm according to manufacturer’s instructions (Abcam).

### Calcium flux in ASM in *hORMDL3*^Myh11eGFP-cre^ mice

To determine calcium flux, 2 × 10^5^ ASM cells/well from WT and *hORMDL3*^Myh11eGFP-cre^ mice were seeded overnight in a 96-well plate in media containing 1 mM extracellular Ca^2+^. Calcium flux experiments were performed in minimum essential medium in the presence or absence of 1 mM extracellular Ca^2+^. Twenty-four hours later, the ASM cells were loaded with the calcium-sensitive Fluo-4 Direct dye reagent and incubated at 37°C for 60 minutes according to manufacturer’s instruction (Invitrogen, Thermo Fisher Scientific). Fluorescence intensity of cultured ASM cells was then measured to determine both the baseline unstimulated level and the level following histamine stimulation (100 μM) with spectrophotometry setting for excitation at 494 nm and emission at 516 nm. Fluorescence intensity at baseline and following histamine stimulation were both measured every 20 seconds for 4 minutes.

### Quantitation of ASM, peribronchial fibrosis, and mucus in lungs of *hORMDL3*^Myh11eGFP-cre^ mice

Lungs from naive *hORMDL3*^Myh11eGFP-cre^ and naive WT mice aged 12 weeks (*n* = 12 mice/group) were processed for immunohistology (paraffin-embedded lung sections) as previously described in this laboratory (8). As naive universal *hORMDL3*-Tg mice develop spontaneous increased ASM, peribronchial fibrosis, mucus, and AHR in the absence of airway inflammation by 12 weeks of age (8), this same 12-week time point was chosen to study naive mice selectively expressing *hORMDL3* in ASM. For paraffin-embedded sections, the left lung was inflated with an intratracheal injection of the same volume of 4% paraformaldehyde solution (Sigma Chemicals) to preserve the pulmonary architecture. Additionally, right lung lobes were initially snap-frozen in liquid nitrogen and stored at –80°C for later processing for protein and RNA extraction.

#### Quantitation of ASM.

Lung sections were immunostained with an anti–alpha–smooth muscle actin primary antibody (Abcam; catalog number ab7817; clone 1A4) to detect peribronchial smooth muscle. Quantification of the positively stained peribronchial area in paraffin-embedded lung sections was outlined and measured using a light microscope (Leica DMLS, Leica Microsystems) attached to an image analysis system (Image-Pro Plus, Media Cybernetics) as previously described (8). Results are expressed as the area of peribronchial alpha–smooth muscle actin staining per micrometer length of basement membrane of bronchioles 150–200 μm internal diameter.

#### Quantitation of peribronchial fibrosis.

The area of peribronchial trichrome staining in paraffin-embedded lungs was outlined and quantified under a light microscope (Leica DMLS, Leica Microsystems) attached to an image analysis system (Image-Pro Plus). Results are expressed as the area of trichrome staining per micrometer length of basement membrane of bronchioles 150–200 μm.

#### Mucus.

To determine the level of mucus expression in the airway, the numbers of periodic acid–Schiff–positive (PAS-positive) and PAS-negative epithelial cells in individual bronchioles were counted as previously described in this laboratory (60).

### Quantitation of airway inflammation in BAL in *hORMDL3*^Myh11eGFP-cre^ mice

BAL fluid was collected from 12-week-old naive *hORMDL3*^Myh11eGFP-cre^ and naive WT mice by lavaging the lung with 1 mL PBS via a tracheal catheter as previously described (8). BAL fluid was centrifuged and the cell pellets were resuspended with 1 mL of PBS to make Wright Giemsa–stained cytospin slides for total and differential cell counts.

### AHR to MCh in *hORMDL3*^Myh11eGFP-cre^ mice

To determine whether expression of *hORMDL3* in ASM influenced spontaneous AHR to MCh, AHR was assessed in naive *hORMDL3*^Myh11eGFP-cre^ and naive WT mice at 12 weeks of age (*n* = 12 mice/group). Mice were anesthetized with ketamine (100 mg/kg) and xylazine (10 mg/kg) i.p., intubated, and ventilated (FlexiVent ventilator; Scireq) as previously described (8). The dynamic airway resistance and elastance were determined using Scireq software in mice exposed to nebulized PBS and methacholine doses at 0, 3, 24, and 48 mg/mL. Ventilator settings include tidal volume set at 10 mL/kg, frequency at 150/min, and positive end-expository pressure at 3 cmH_2_0.

### Acute HDM challenge

WT and *hORMDL3*^Myh11eGFP-cre^ mice aged 12 weeks were administered intranasal *Dermatophagoides pteronyssinus* extract (Greer Laboratories) at 100 μg on days 0, 7, 14, and 21 as previously described (61). On day 24 mice had AHR to MCh assessed and were sacrificed (61). Control groups of WT and *hORMDL3*^Myh11eGFP-cre^ mice aged 12 weeks were not administered HDM.

### Statistics

All results are presented as mean ± SEM. A statistical software package (GraphPad Prism) was used for the analysis. A 2-tailed *t* test was used for analysis of 2 groups. One-way ANOVA was used when more than 2 groups were compared. *P* values of less than 0.05 were considered statistically significant.

### Study approval

The acquisition of deceased human lung donor tissue was reviewed by the University of Arkansas for Medical Sciences Institutional Review Board and determined not to be human subject research. The mouse studies were approved by the University of California, San Diego, Institutional Animal Care and Use Committee.

## Author contributions

AKP designed and performed the experiments, analyzed the data, and prepared the figures. MM, PR, SD, NW, and SJ assisted AKP in performing experiments. JB and TAD assisted AKP in performing the FACS studies. RCK provided postmortem asthma lungs for ASM studies and provided feedback on the manuscript. BO reviewed ASM studies and provided feedback on the manuscript. DHB conceived the study, obtained funding for the study, designed experiments, supervised the work, and wrote the manuscript with AKP.

## Supplementary Material

Supplemental data

## Version 1 03/04/2021

In-Press Preview

## Version 2 04/08/2021

Electronic publication
