# Supplementary material for: ORMDL3 expression in ASM regulates hypertrophy, hyperplasia via TPM1 and TPM4, and contractility
Source: JCI Insight. 2021 Apr 8;6(7):e136911. doi: 10.1172/jci.insight.136911 (PMC8119187; doi:10.1172/jci.insight.136911)
Supplement: Supplemental data [file jciinsight-6-136911-s035.pdf]

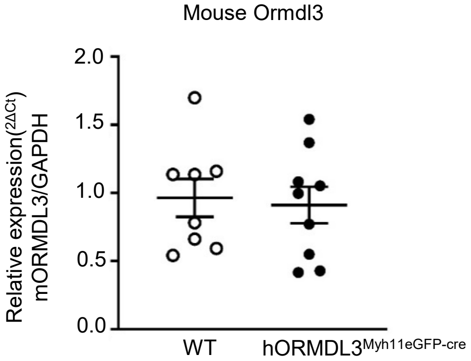

Supplement Figure 1. Mouse Ormdl3 expression in airway smooth muscle

Levels of Ormdl3 mRNA were quantitated by RT-qPCR in ASM derived from WT and hORMDL3$^{Myh11eGFP-cre}$ mice (n=8-9/group). P-values are results of unpaired t-tests.

A

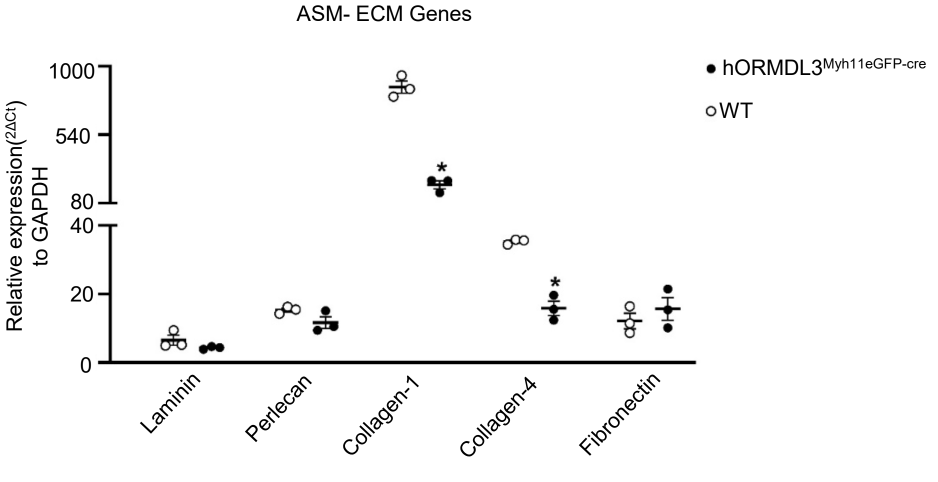

B

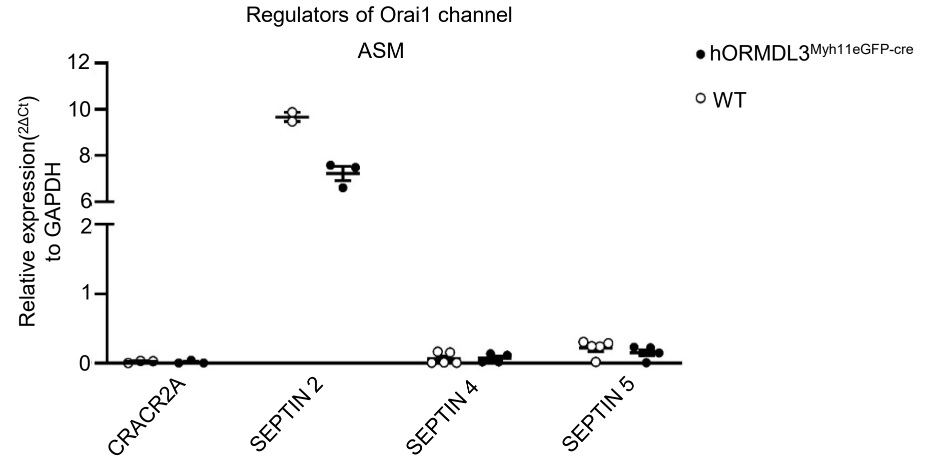

Supplement Figure 2. Effect of ORMDL3 on ASM expression of extracellular matrix (ECM) and CRAC channel regulator mRNA.
mRNA expression of (A) ECM genes including laminin, perlecan, collagen-1, collagen-4, and fibronectin (n=3-5/group), as well as (B) regulators of the CRAC channel Orai1 including CRACR2A, Septin 2, Septin 4 and Septin 5 were assessed in ASM from WT and hORMDL3$^{Myh11eGFP-cre}$ mice (n=3-5/group). * $p < 0.05$ compared to wildtype. P-values are results of unpaired t-tests.

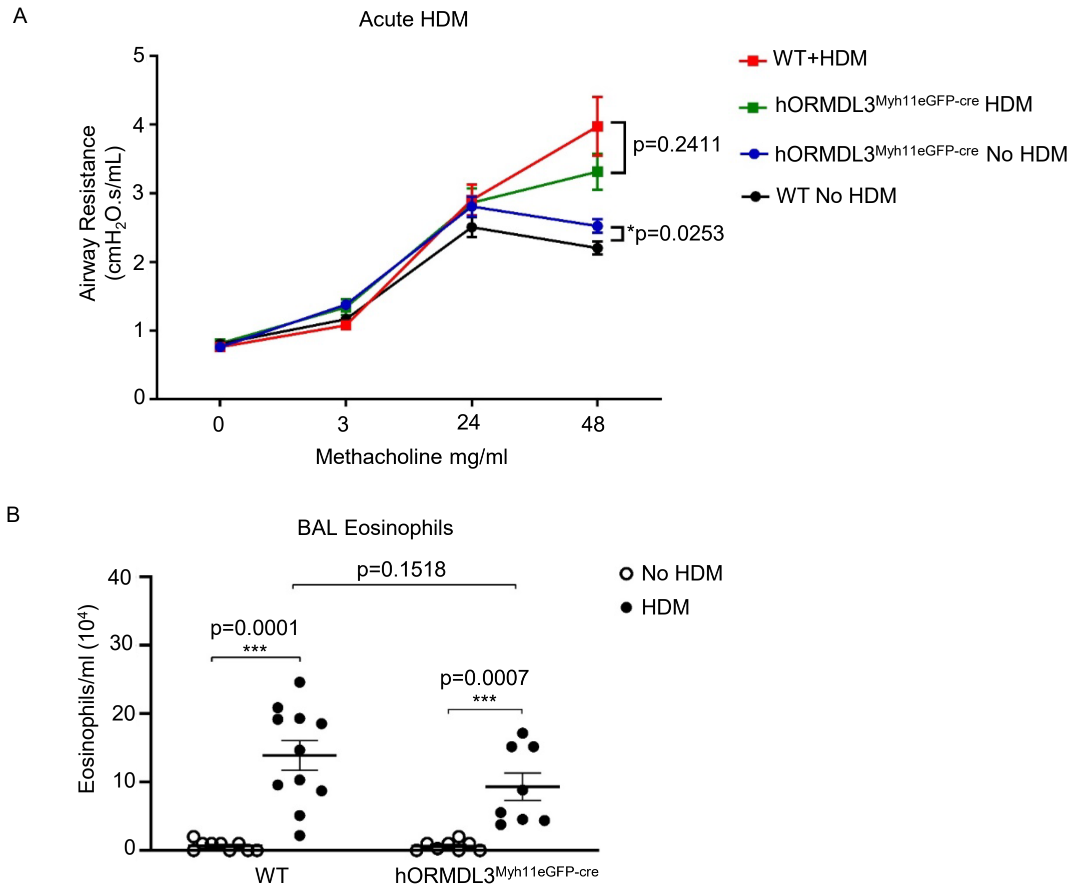

Supplement Figure 3. Effect of ORMDL3 on AHR and BAL eosinophils following HDM allergen challenge. Twelve weeks old wildtype (WT) and hORMDL3$^{Myh11eGFP\text{-}cre}$ mice were challenged with HDM intranasally at day 0, 7, 14, and 21. Mice were sacrificed on day 24. (A) Airway resistance following methacholine challenge was assessed in intubated mice (n=9-11 mice/group). (B) BAL eosinophils were determined by Wright Giemsa quantitation of BAL eosinophils in WT and hORMDL3$^{Myh11eGFP\text{-}cre}$ mice (n=9-11 mice/group). * $p < 0.05$ compared to wildtype. P-values are results of two-way ANOVA with Tukey's post-hoc test.
